# Deciphering the Polymorphism of CaSi_2_: The Influence of Heat and Composition

**DOI:** 10.1021/acs.inorgchem.4c00902

**Published:** 2024-05-24

**Authors:** Xian-Juan Feng, Wilder Carrillo-Cabrera, Alim Ormeci, Mitja Krnel, Ulrich Burkhardt, Bodo Böhme, Yuri Grin, Michael Baitinger

**Affiliations:** Max-Planck-Institute for Chemical Physics for Solids, 01187 Dresden, Germany

## Abstract

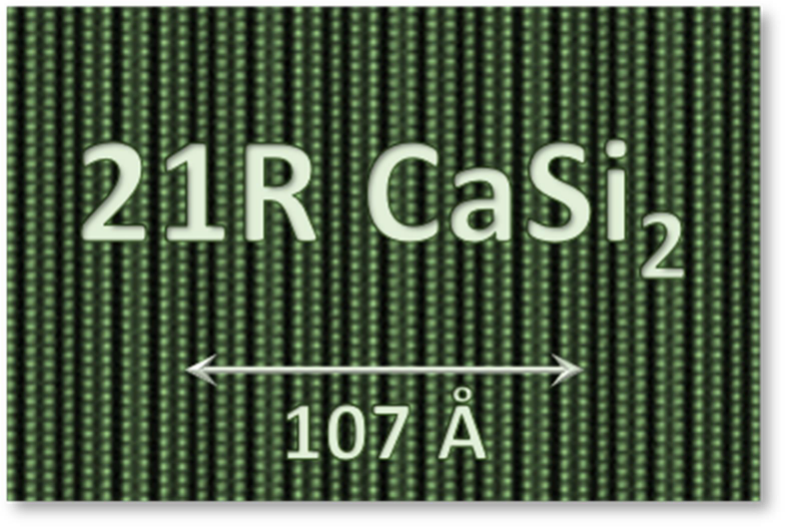

The Zintl phase CaSi_2_ is a layered compound with stacking variants known as 1P, 3R, and 6R. We extend the series by the 21R polytype formed by rapid cooling of the melt. The crystal structure of 21R-CaSi_2_ (space group *R*3̅*m*) was derived from HRTEM images, and the atomic positions were optimized by using the FPLO code (*a* = 3.868 Å, *c* = 107.276 Å). We explore polytype transformations by powder X-ray diffraction (PXRD), transmission electron microscopy (TEM), scanning electron microscopy (SEM), electron backscattering diffraction (EBSD), and thermal analysis. While 6R-CaSi_2_ is thermodynamically stable at ambient conditions, nanosized impurities of silicon stabilize 3R-CaSi_2_ as a bulk phase.

## Abstract 2

The marginal enthalpy difference between 3R and 6R-CaSi_2_ brings entropy into play, enabling intriguing ordering variants. This article presents the new modification 21R-CaSi_2_, which can be regarded as an intergrowth of the 3R and 6R-CaSi_2_ structures, along with an exploration of the relationship between the known polytypes.

## Introduction

**Figure 1:**
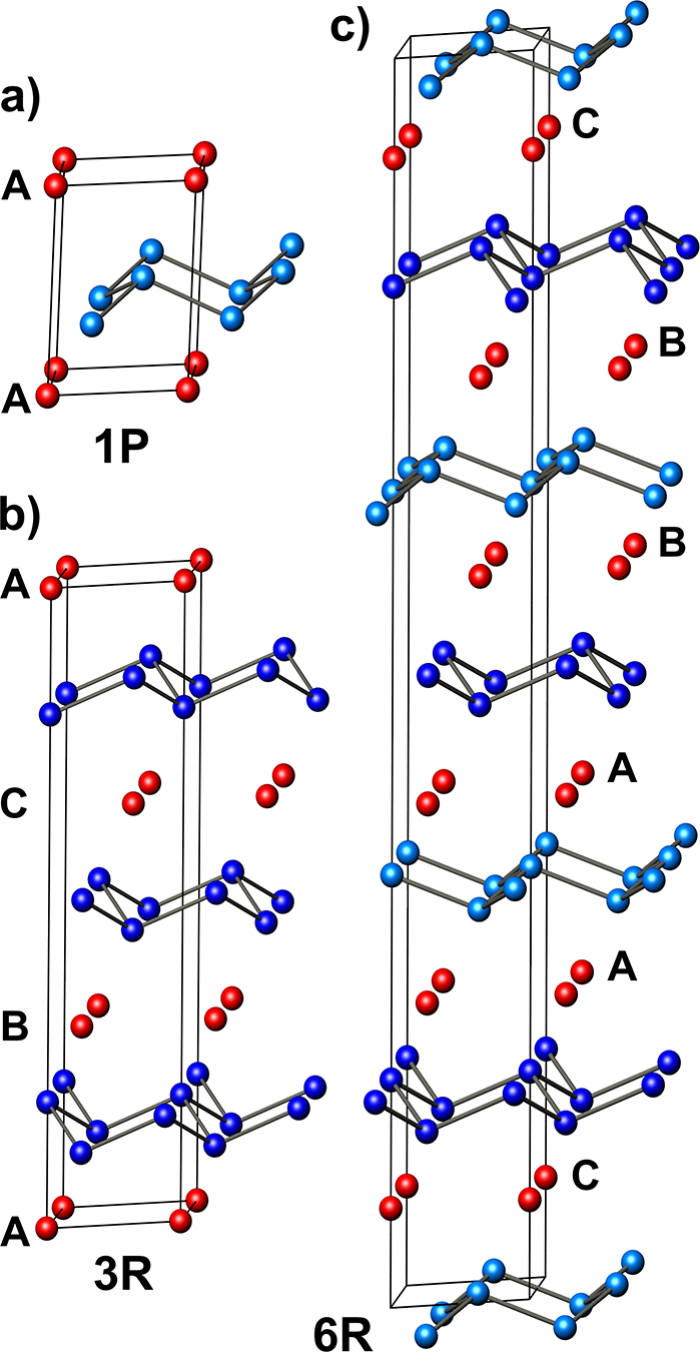
Known polytypes of CaSi_2_: (a) 1P-CaSi_2_ with one Si layer per unit cell (space group *P*3̅*m*1); (b) 3R-CaSi_2_ with three; and (c) 6R-CaSi_2_ with six silicon layers per unit cell (both *R*3̅*m*). Ca atoms are drawn red, and Si atoms are blue. A, B, and C are registry letters of the Ca layers.

CaSi_2_ is utilized in various applications, serving as a deoxidizer and desulfurizer in steel production,^1^ as well as a reactive primer in firearm cartridges.^2^ In the field of material research, the significance of CaSi_2_ lies in its crystal structure, which features infinite 2D silicon layers.^3^ These layers can be detached in acidic aqueous solutions, enabling the synthesis of OH-functionalized siloxenes Si_6_O_*x*_H_*x*_,^4,5^ which have also been prepared directly via the oxidation of epitaxial films.^6^ Furthermore, via low-temperature reactions, the silicon monolayers have been protonated to form polysilane (Si_6_H_6_)_*n*_,^7^ opening up possibilities for the utilization of 2D silicon nanomaterials in electronic devices.^8^ The crystal structure of CaSi_2_ is known for three stacking variants 1P, 3R, and 6R.^9,10^ In all variants, the three-bonded silicon anions (3b)Si^–^ form infinite puckered 2D layers reminiscent of those found in gray arsenic or the (111) surface plane of α-Si. The Ca cations separate these anionic silicon sheets (Figure 1). Although all CaSi_2_ polytypes are 3D metals with complex chemical bonding,^11^ the connectivity of silicon and the electron-balance Ca^2+^(3b)(Si^–^)_2_ are indicative of a Zintl phase. The simplest stacking variant, 1P-CaSi_2_, a superconductor with a critical temperature of 14 K, is only stable under high-pressure conditions.^12,13^ At room temperature and increased pressure, 1P-CaSi_2_ transforms into an AlB_2_-type modification,^13^ while high-pressure high-temperature conditions lead to a tetragonal ThSi_2_ type of structure.^14^ At ambient pressure, free energy calculations have demonstrated that the 6R-CaSi_2_ polytype is thermodynamically stable.^9^ However, the typical synthesis methods for CaSi_2_ often yield mixtures of the 3R and 6R polytypes, leading to inconsistencies in the results obtained from different studies. Single-phase 6R-CaSi_2_ has been successfully obtained through the rapid cooling of stoichiometric melts.^9,15,16^ Furthermore, single-crystal growth of 6R-CaSi_2_ has also been reported using the floating zone method with cooling rates of 10 K/h.^17^ In contrast, the formation of 3R-CaSi_2_ is favored when slow cooling rates have been applied.^16^ The discrepancy with the thermodynamic calculations is notable, as the metastable polymorph should, at best, form through fast cooling. Additionally, the formation of 3R-CaSi_2_ has also been observed after annealing of 6R-CaSi_2_ at various temperatures between 200 and 800 °C under 20 bar H_2_ pressure.^9^ Remarkably, the resulting 3R-CaSi_2_ did not transform back to stable 6R-CaSi_2_ upon further annealing under vacuum, which contradicts the expectations of a metastable state.

The influence of impurities on the phase formation has been the subject of several studies. It has been suggested that Sr impurities stabilize the 6R-CaSi_2_ polytype,^18^ while the addition of LiF triggers the transformation to 3R-CaSi_2_.^16^ Moreover, annealing 6R-CaSi_2_ single crystals at 800 °C in an evacuated glass ampule has yielded single-phase 3R-CaSi_2_, whereas no transformation was observed in a closed Ta ampule.^19^ Another remarkable finding is the transformation of 6R-CaSi_2_ into 3R-CaSi_2_ at low temperatures in ionic liquids (ILs).^16^ This caught our attention, as our research project also aims to convert reactive Zintl phases in ionic liquids to metastable compounds (e.g., Zn_2_Si_5_) or allotropes (e.g., Ge *cF*136).^20−23^ After a treatment of 6R-CaSi_2_ in the IL DTAC/AlCl_3_, we also observed the conversion of 6R-CaSi_2_ to well-crystalline 3R-CaSi_2_. The required temperature of no more than 80 °C seemed surprisingly low to us, and we decided to examine the transformation process of the CaSi_2_ polytypes in greater detail.

## Experimental Section

### Preparation of As-Cast 6R-CaSi_2_

**Figure 2:**
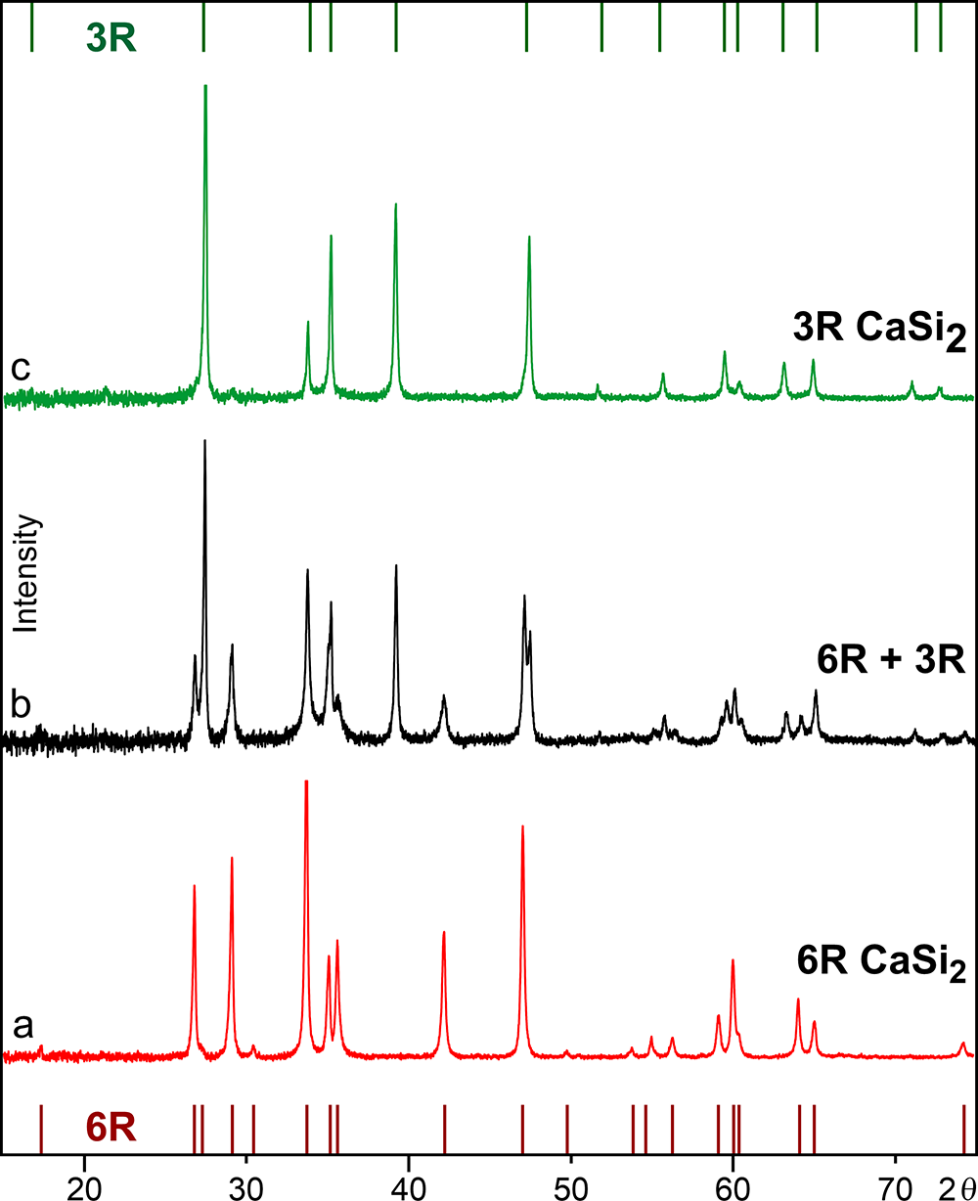
PXRD patterns tracing the transformation from rapidly cooled 6R-CaSi_2_ to 3R-CaSi_2_ (Cu Kα_1_ radiation). (a) 6R-CaSi_2_ after rapid quenching; (b) a partially converted specimen; and (c) 3R-CaSi_2_ after complete conversion.

The preparation was carried out in an Ar-filled glovebox. A stoichiometric mixture of Ca (99.9%, dendritic pieces, Chempur) and Si (5N, granulated, Chempur) was loaded into a glassy carbon crucible and melted using induction heating. The melt was then poured onto a steel plate and rapidly cooled down to room temperature by punching the droplet with a second plate.^24^ This process yielded ≈2 g of 6R-CaSi_2_ in the form of thin platelets (Figure S1). Powder X-ray diffraction (PXRD) and scanning electron microscopy (SEM) analyses did not show any impurity phases in the product (Figures 2a, S2, and S3). In samples subjected to prolonged heating in open crucibles at 900 °C or higher, small amounts of α-Si were detected due to the evaporation of Ca. Consequently, if one were to prepare samples in this manner, they would contain less Ca.

### Annealing Experiments

The annealing experiments were conducted using freshly prepared samples of active 6R-CaSi_2_ in Nb ampules (*d* = 5 mm, *l* = 15 mm). For annealing at 900 °C, the ampules were closed by arc welding to prevent evaporation. PXRD analysis of the products obtained after reaction times up to 2 days solely showed reflections of 6R-CaSi_2_. For experiments at 600 °C, the ampules were mechanically sealed to avoid uncontrolled heating of the sample during the welding process. For annealing, the tantalum ampules were placed inside Schlenk vessels, which were subsequently loaded into a vertical tube furnace. The tube furnace was situated within an argon-filled glovebox. Specimens were periodically removed from the furnace for ex-situ analysis via PXRD, with intervals of every hour up to 8 h and every day up to 5 days. The obtained products exhibited varying amounts of 3R-CaSi_2_ and 6R-CaSi_2_ depending on the annealing time.

### Oxidation by AlCl_3_

Five mL of a 0.03 M solution of AlCl_3_ (sublimed) in toluene was heated to 80 °C under an Ar atmosphere. The solution was stirred with a glass-protected stir bar, and a platelet of rapidly quenched 6R-CaSi_2_ was added. After 4 h, the piece was removed, washed with toluene, and dried under Ar. PXRD analysis revealed equal amounts of 3R- and 6R-CaSi_2_ in the sample (Figure S14). SEM analysis did not detect any Al in the CaSi_2_ grains. Pure toluene had no effect on 6R-CaSi_2_.

### Reaction in Hydrogen Plasma

Twenty mg of finely powdered 6R-CaSi_2_ were loaded into an Al_2_O_3_-crucible (*d* = 10 mm, *l* = 15 mm). The crucible was then placed inside a quartz glass reactor (*d* = 25 mm, *l* = 30 cm) and positioned within a microwave furnace (MLS, Pyro). The plasma was ignited at 800 W in a H_2_-stream, maintaining a total pressure of 5 mbar. After 30 min of reaction time, 50% of the sample had converted to 3R-CaSi_2_.

### PXRD

PXRD patterns were recorded on a Guinier camera (Huber G670, image plate detector, Cu *K*α_1_ radiation, Ge(111) monochromator, 5° ≤ 2 θ ≤ 100°, Δ2θ = 0.005°). The specimens were finely ground in an argon-filled glovebox. The powders were fixed on the sample holder between two polyimide foils (7.5 μm, Kapton, Chemplex) using a thin film of vacuum grease (Lithelen, Leybold) as an adhesive. Lattice parameters were refined with internal standard LaB_6_ (NIST, SRM 660a, *a* = 4.1569162(97) Å), which was admixed to the sample before the PXRD measurement.^25^ An elevated background in the diffraction patterns, occurring independently of the sample at low diffraction angles and thus caused by the experimental setup, was subtracted.

### Differential Thermal Analysis (DTA)

Specimens of about *m* = 30 mg were filled in glassy carbon crucibles (Sigradur, HTW), which were welded in Nb crucibles. The measurements were performed with a heat-flux DTA device (Netzsch DSC 404C) at a constant heating rate of 10 K/min.

### SEM

Investigations were performed using a field-emission scanning electron microscope (FE-SEM, JEOL, JSM-7800F) equipped with detectors for backscattered and secondary electrons. A silicon drift detector (SDD, xflash detector 6/30, Bruker Nano, Berlin) for energy-dispersive X-ray spectroscopy (EDXS) and an electron backscatter diffraction (EBSD) detector were attached to the SEM. Spectra and Kikuchi pattern recording and evaluation are realized with the combined Esprit Quantax 400 and Esprit crystAlign 400 systems (Bruker Nano, Berlin). EBSD patterns were calculated using the software module Esprit DynamicS Version 2.2.

### Transmission Electron Microscopy (TEM)

The specimens for the TEM investigation (lamellar cross sections) were prepared with the focused ion beam (FIB) technique using a Quanta 200 3D ion/electron dual-beam device (FEI, Eindhoven) equipped with an Omniprobe micromanipulator (W needle), which can be used as a scanning electron microscope and a scanning ion microscope. The FIB lamellar samples (30 to 40 nm thin) were investigated by TEM (conventional TEM and HRTEM) and selected area electron diffraction (SAED). The TEM investigations were performed on a FEI Tecnai F30-G2 supertwin microscope operating at 300 kV equipped with a CCD camera (GATAN Inc.) and a standard double-tilt holder (GATAN Inc.).

### Magnetic Susceptibility

Magnetic properties in the temperature range 1.8 K ≤ *T* ≤ 350 K were measured on thin plates (*m* = 45 mg) in a silica tube using a SQUID magnetometer (MPMS XL-7, Quantum Design) at external fields between 2 mT and 7 T. The raw data obtained for the magnetic susceptibility were corrected for the diamagnetic contribution of the silica tube, which had been determined beforehand. To account for small ferromagnetic impurities in the range of few ppm (calculated for pure iron), which probably originate from steel tools applied during sample preparation, data sets measured at 3.5 and 7 T were used to perform an extrapolation to an infinite external field by the Honda-Owen method.^26^

### Calculations

The first-principles electronic structure calculations were carried out with the all-electron full-potential local orbital (FPLO) method.^27^ In the scalar relativistic calculations, the exchange-correlation effects were considered by the generalized gradient approximation (GGA) to the density functional theory as parametrized by Perdew et al.^28^ The crystal structures of 3R, 6R, and 21R-CaSi_2_ were each fully optimized at selected volumes to obtain equations of state, *E*(*V*). The Brillouin zones of the rhombohedral unit cells were sampled by meshes of 21^3^, 18^3^, and 12^3^, respectively. The maximum force on an atom to stop the optimization procedure was chosen as 5.0 meV Å^–1^. The resulting *E*(*V*) values were fitted by fourth-order polynomials using a Birch–Murnaghan equation of state.^29,30^ The pressure, *p*, was computed from the volume derivative of the fitted curves so that 0 K enthalpies could be obtained as *H*(*p*) = *E*(*V*[*p*]) + *p*·*V*(*p*). The crystal structures of the elemental solids, fcc Ca and α-Si, were also optimized in order to calculate the formation enthalpies of the compound. Chemical bonding analysis in position space was conducted using the electron localizability approach.^31−35^ This approach involves calculating the electron density (ED) and electron localizability indicator (ELI) on a uniform grid in position space using a module implemented in the FPLO package.^36^ The program DGrid was utilized for the topological analysis of ED and ELI.^37^ The basin intersection method^38^ was then employed to determine the individual atom contributions, in terms of electron counts, to the bonds.

### Safety Statement

No uncommon hazards are noted.

## Results and Discussion

### As-Cast 6R-CaSi_2_

**Figure 3:**
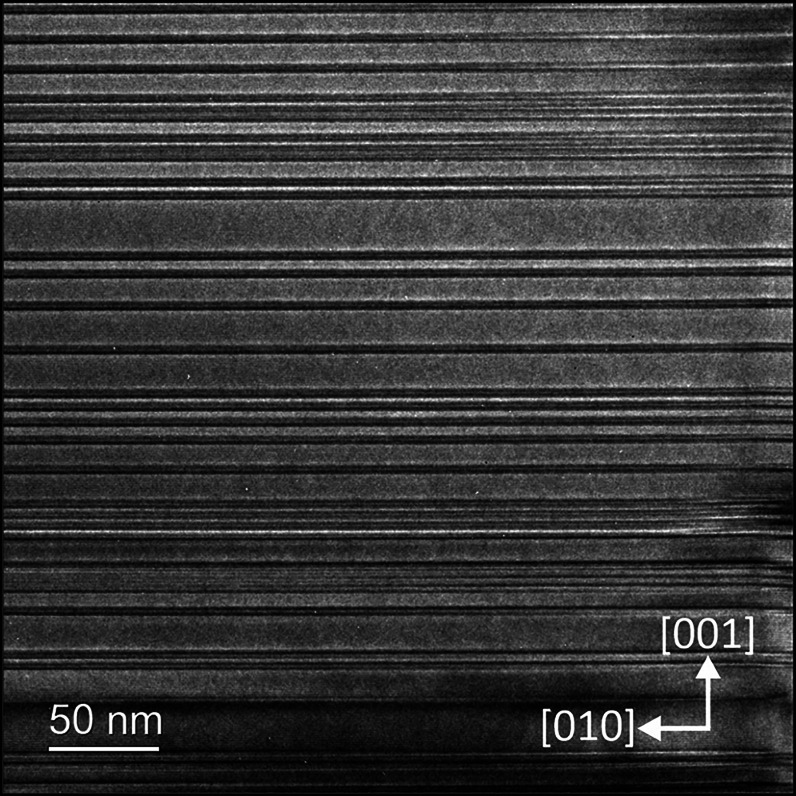
HRTEM image of 6R-CaSi_2_ obtained from rapid quenching shows a remarkably high density of stacking faults perpendicular to [001].

**Figure 4:**
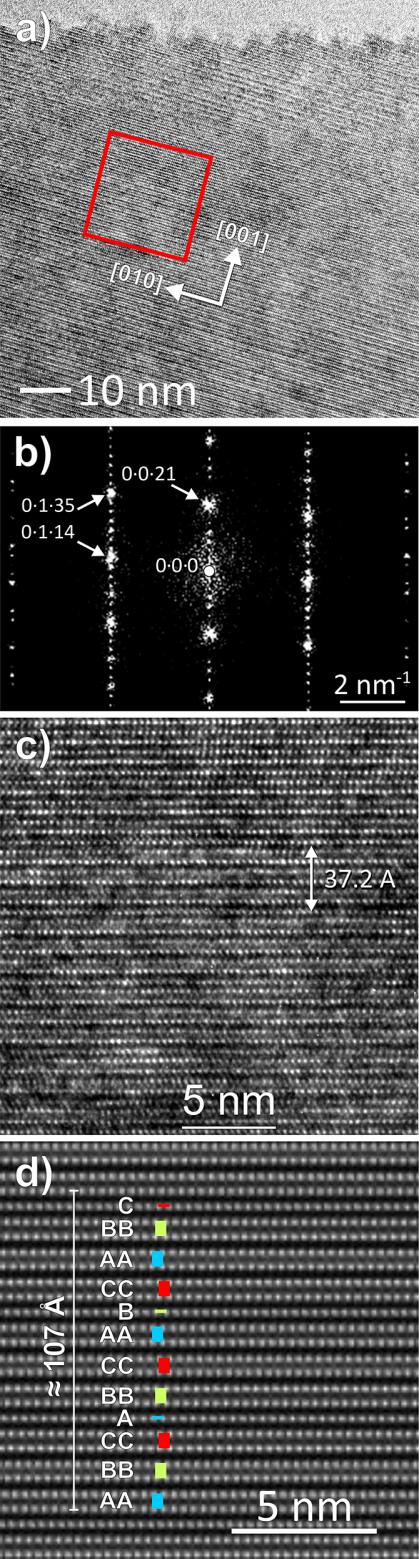
(a) HRETM images of a subsurface region, white spots are Ca atoms; (b) SAED pattern conforming the unit cell; (c) red square region of (a) in higher resolution; and (d) Bragg-filtered image indexed with registry letters A, B, and C of the Ca atoms.

Our first objective was to develop a straightforward process for producing bulk quantities of a single-phase CaSi_2_ modification. We finally obtained 6R-CaSi_2_ using a rapid cooling technique^24^ applied to a stoichiometric melt. The product was single-phase as confirmed by PXRD analysis (Figure 2a) and SEM (Figures S2 and S3), and the lattice parameters of different batches closely matched literature values (Table 1). The result aligns with previous studies stating that rapid cooling favors the formation of 6R-CaSi_2_.^16^ Conventional optical microscopy images initially showed a homogeneous product as well, but under polarized light, distinct stripes emerged on the surface of the grains (Figure S3). Such features are typically indicative of crystal defects, including twinning, antiphase boundaries, or stacking faults of the 2D layers. It is plausible that these defects resulted from the high cooling rate applied. EDX spectroscopy consistently confirmed the expected chemical composition, and EBSD analysis validated the presence of 6R-CaSi_2_ across the entire sample surface (Figure S6a). To further investigate the morphology, thin lamellas were cut from the sample surface using the FIB technique and then analyzed using high-resolution transmission electron microscopy (HRTEM). The HRTEM images unveiled a significant concentration of stacking faults along the [001] direction (Figure 3), while still displaying regular SAED patterns characteristic of 6R-CaSi_2_ (Figure S8b). In some regions of the particles, the density of stacking faults further increased, manifesting now in SAED as diffuse intensity lines that connect the diffraction spots of the 6R lattice (Figure S8c). Within isolated areas, these stacking faults condense to the 21R-CaSi_2_ structure (Figure 4a–c). Notably, the concentration of the new polytype fell below the detection limit of PXRD analysis. A preliminary structure model of 21R-CaSi_2_ was derived from HRTEM images, which revealed a lattice parameter *c* ≈ 107 Å (Figure 4d). Subsequently, the model was optimized using the FPLO method,^27^ as described in detail below.

**Table 1:** Lattice Parameters and Unit Cell Volume of 3R- and 6R-CaSi_2_ Obtained with Different Heat Treatments

CaSi_2_		*a* [Å]	*c* [Å]	*V* [Å^3^]
6R	quenched	3.8561(4)	30.643(5)	394.60(9)
		3.8573(4)	30.626(4)	394.62(8)
	annealed 900 °C	3.8563(5)	30.652(9)	394.8(1)
3R	annealed 600 °C	3.8253(3)	15.882(1)	201.26(2)
		3.8264(1)	15.8888(9)	201.47(1)

### Transformation by Annealing

The high defect concentration observed in 6R-CaSi_2_ after rapid cooling indicates that these samples are in a nonequilibrium state at room temperature. This led us to the question of whether these samples would undergo a transformation during subsequent heat treatment. All annealing experiments were terminated by quenching the ampules in water to minimize the influence of the cooling process on the phase composition. When as-cast 6R-CaSi_2_ was annealed in this way at 900 °C, PXRD measurements yielded consistent results before and after annealing (Table 1). In contrast, the product obtained at 600 °C showed a strong dependence on the annealing time (Figure S13). From 3 h onward, the products contained an increasing proportion of the 3R type. After 12 h, a nearly equimolar mixture of 3R and 6R-CaSi_2_ had formed, and after 20 h, 3R-CaSi_2_ was the majority phase (Figure 2b). TEM images of the 20 h sample revealed a matrix of 3R-CaSi_2_ enclosing isolated slabs of 6R-CaSi_2_ (Figure S9a). Both phases of the specimen displayed regular SAED patterns (Figures S9b and S10). Surprisingly, EBSD analysis of the sample surface solely detected 3R-CaSi_2_ (Figure S6b). Faster than in the bulk phase, the transformation from 6R-CaSi_2_ to 3R-CaSi_2_ was already completed on the surface of the platelets. After 2 days of annealing, a single-phase bulk sample of 3R-CaSi_2_ was obtained according to PXRD (Figure 2c). In this case, however, the surface of the sample differed from the bulk phase as well. EBSD images of the sample surface indicated 30% 6R-CaSi_2_ and 70% 3R-CaSi_2_ (Figure S6c). With longer annealing times, the proportion of 6R-CaSi_2_ increased, both at the surface and in the bulk sample, and after 3–5 days at 600 °C, the products predominantly consisted of 6R-CaSi_2_ again. The lattice parameters of both modifications did not vary during the transformation process. The experiments suggest that 3R-CaSi_2_ forms as an intermediate phase during the recrystallization process of 6R-CaSi_2_ samples with high defect concentrations.

The reaction times specified above strongly depend on the morphology of the starting material. They were most reproducible for platelet-shaped samples of 6R-CaSi_2_ that were used immediately after rapid cooling. Two influencing factors on the reaction time could be identified:

- **Particle size:** Samples of defect-rich 6R-CaSi_2_ ground under Ar to a particle size of 20 μm did not transform over a period of 5 days.
- **Composition:** With an excess of calcium at the nominal composition Ca_1.17_Si_2_, rapid quenching of the melt resulted, as expected from the phase diagram,^39^ in a mixture of Ca_14_Si_19_ and 6R-CaSi_2_. Even after 2 days annealing at 600 °C, no traces of the 3R phase were detected in the PXRD analysis. Contrarily, an excess of α-Si did not hinder the conversion.

The observations suggest that α-Si seeds are required to transform 6R-CaSi_2_ to 3R-CaSi_2_. Starting from stoichiometric samples, small amounts of α-Si can form, e.g., by evaporation of calcium or through oxidation. Indeed, we have found silicon double layers with HRTEM in 3R-CaSi_2_ samples that may absorb an excess of silicon. While not detectable by PXRD, such 2D defects still allow the growth of large crystalline regions of 3R-CaSi_2_ (Figure S11b). We assume that 3R-CaSi_2_ samples become stabilized when the concentration of finely distributed α-Si exceeds a certain threshold (see below). This model would explain the observation by Nedumkandathil et al., who reported that 3R-CaSi_2_ samples prepared under H_2_ pressure do not undergo a transformation into the thermodynamically stable 6R-CaSi_2_.^9^ The influence of composition might also explain the experiment of Castillo et al.,^19^ who could transform 6R-CaSi_2_ to 3R-CaSi_2_ by annealing in glass, but not in Ta ampules. In Ta ampules, the formation of small amounts of TaSi_2_ may ensure an excess of Ca in the sample, blocking the transformation to 3R-CaSi_2_. In contrast, annealing in glass ampules may lead to an excess of α-Si by the formation of CaO.

### Oxidation Reactions

The influence of oxidizing agents on the transformation of 6R-CaSi_2_ to 3R-CaSi_2_ was investigated using two experimental setups. In the first experiment, a platelet of as-cast 6R-CaSi_2_ was subjected to treatment in dry toluene at 80 °C. The treatment had no effect on the specimen according to PXRD. Then, the experiment was performed with a 0.03 M solution of AlCl_3_ in toluene at the same temperature. After 4 h, the surface of the platelet exhibited brown tarnish, while the interior remained metallic gray. From PXRD, the bulk sample had converted by 50% to 3R-CaSi_2_ (Figure S14). α-Si and Al were not detected in the CaSi_2_ grains by SEM. We conclude that the Al^3+^ cations oxidize the Si^–^ anions of CaSi_2_ at the grain surface to Si^0^, which triggers the conversion process.

In a second experiment, 20 μm powder of as-cast 6R-CaSi_2_ was filled in a Duran glass crucible and treated in a microwave-induced H_2_-plasma (*p*(H_2_) = 5 mbar). As mentioned above, powder samples from the same batch did not undergo conversion under an Ar atmosphere at 600 °C. Upon plasma treatment at a power of 800 W, the temperature of the glass vessel reached approximately 200 °C. After a reaction time of 30 min, about 50% of the sample had converted to 3R-CaSi_2_. We conclude that either the Si^–^ anions were oxidized by the plasma at the particle surface to Si^0^ or Ca evaporated. In both scenarios, the transformation could be triggered by α-Si. However, the content of α-Si in the products was below the detection limit of PXRD.

### Phase Relations in the System Ca/Si

Differential scanning calorimetry (DSC) performed on single-phase 6R-CaSi_2_ and 3R-CaSi_2_ samples did not reveal any thermal effect indicative of a conversion between the polytypes. In both cases, the peritectic reaction CaSi_2_ → Ca_14_Si_19_ + L was observed at 1045(5) °C, which supports the suggested phase diagram by Heyrman and Chartrand.^39^ The eutectic melting of CaSi_2_ and α-Si was measured at 1028(5) °C. Since CaSi_2_ forms incongruently, the formation of a single-phase product from the melt was unexpected. However, it was previously assumed from crystal growth experiments that the undercooled melt could reach the stability range of CaSi_2_.^17^ Interestingly, the formation of mm-sized single crystals of 6R-CaSi_2_ has also been reported in the ternary systems Ca/Si/O^40^ or in Ta ampules,^41,42^ suggesting further investigations of the respective ternary systems.

### Structural Principles of the CaSi_2_ Polytypes

**Figure 5:**
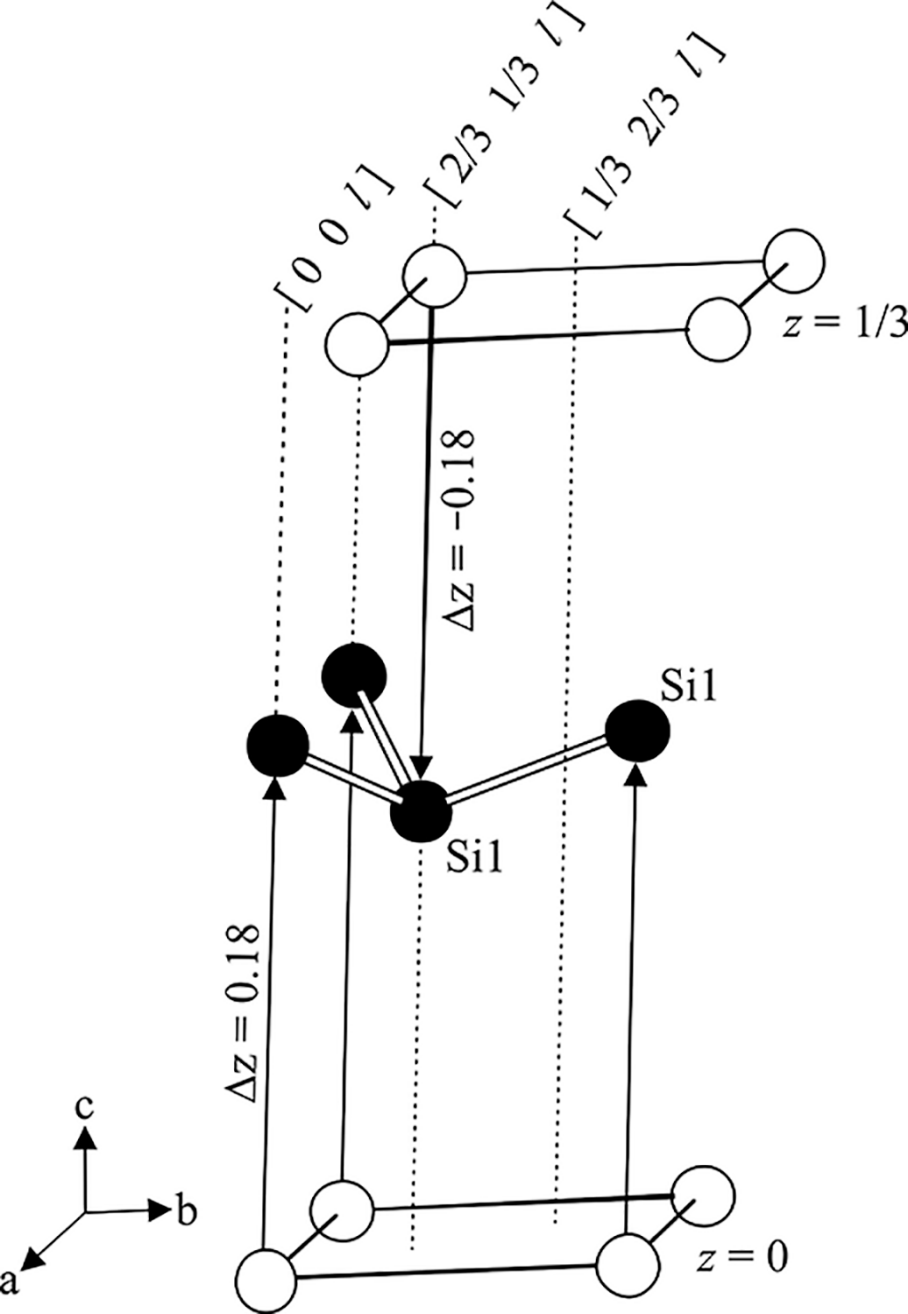
Construction of the puckered Si1 layer in the unit cell of 6R-CaSi_2_ for Si1.

**Figure 6:**
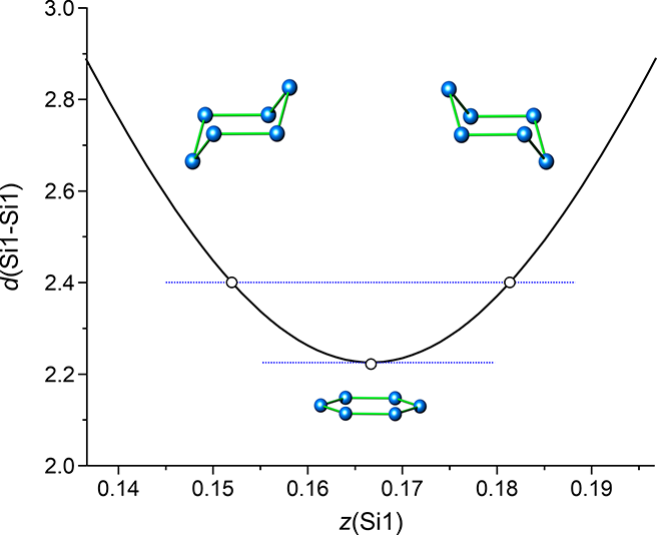
Correlation between the atomic position (0 0 *z*) and the interatomic distance *d*(Si1–Si1). There are two solutions for an ideal distance *d*(Si–Si) = 2.4 Å, *z* = 0.15 or 0.18. Both values represent puckered Si layers inverted to each other.

In the various stacking variants of CaSi_2_, the atoms have different local environments, which can result in significant differences in energy among the variants. In the crystal structures of the known CaSi_2_ modifications, specific Aufbau rules are observed, which are analyzed below and utilized for the structure determination of 21R-CaSi_2_. Both metastable 3R-CaSi_2_ and the thermodynamically stable 6R-CaSi_2_ crystallize in the rhombohedral space group *R*3̅*m* (No. 166). In 6R-CaSi_2_, the unit cell comprises atoms on three crystallographic sites (Ca, Si1, Si2). All atoms occupy Wyckoff positions 6*c*, which are given by (0 0 ±*z*), (2/3 1/3 1/3 ± *z*), and (1/3 2/3 2/3 ± *z*), respectively. This means that all atoms are situated in three columns [0 0 *l*], [2/3 1/3 *l*], and [1/3 2/3 *l*] along the crystallographic *c*-axis. A hypothetical atom placed at the origin of the unit cell (actually, there is none in the crystal structure of 6R-CaSi_2_) generates 2D hexagonal nets at *z* = 0, *z* = 1/3, and *z* = 2/3 (Figure 5; white atoms). When the hypothetical atom is moved upward from the origin to (0 0 *z*_1_), its symmetry-equivalent counterpart of the adjacent column is correspondingly shifted downward to the position (2/3 1/3 1/3 – *z*_1_). Approaching each other, both atoms create the puckered 2D layers observed for the Si atoms (Figure 5, black atoms). Within the layer, the interatomic distance between the Si atoms is thus determined by the *z* coordinate of the Wyckoff site and reaches a minimum at *z*_1_ = 1/6 (Figure 6). Regular distances *d*(Si–Si) are either generated at *z*_1_ = 0.15 or at *z*_1_’ = 0.18. In the crystal structure of 6R-CaSi_2_, only the latter position is realized by Si1. By further increase of the *z* value, the atom on the [0 0 *l*]-column will approach its symmetry equivalent of the [1/3 2/3 *l*]-column, (1/3 2/3 2/3 – *z*), at *z*_2_ = 2/6. Again, two *z*-values result in regular distances *d*(Si–Si), *z*_2_ = 0.31, and *z*_2_’ = 0.35. The Si2 atoms in 6R-CaSi_2_ realize the latter one. In this way, two sets of symmetry-equivalent layers–three Si1 and three Si2 layers–are generated in the unit cell of 6R-CaSi_2_. The Ca atoms constitute plane hexagonal nets (*z* ≈ 1/12) separating the Si1 and Si2 layers. The crystal structure of 3R-CaSi_2_ is organized similarly. The unit cell containing one Ca atom at position 3*a* (0 0 0) and one Si atom at 6*c* is only half as large as in 6R-CaSi_2_ and thus provides space for only three silicon layers.

In solid-state chemistry, polytypes of compounds are generally classified by a registry notation.^43^ For instance, 6R-CaSi_2_ is usually characterized by the stacking sequence AABBCC, where these letters denote the arrangement of the six Si layers within the unit cell relative to each other.^6,14^ However, in this study, we use a notation that includes both Ca and Si atoms.^6^ Ca atoms are represented by the uppercase letters A, B, and C, corresponding to atoms within the columns [0 0 *l*], [2/3 1/3 *l*], and [1/3 2/3 *l*], respectively, while Si atoms are denoted by lowercase letters. By incorporating all atoms in this manner, the stacking order of 6R-CaSi_2_ is delineated by [A *bc* A *ba* B *ca* B *cb* C *ab* C *ac*]. Similarly, the stacking order of 3R-CaSi_2_ is [A *ba* B *cb* C *ac*], and for 1P-CaSi_2_, it is [A *bc* A] (Figure 1).

**Figure 7:**
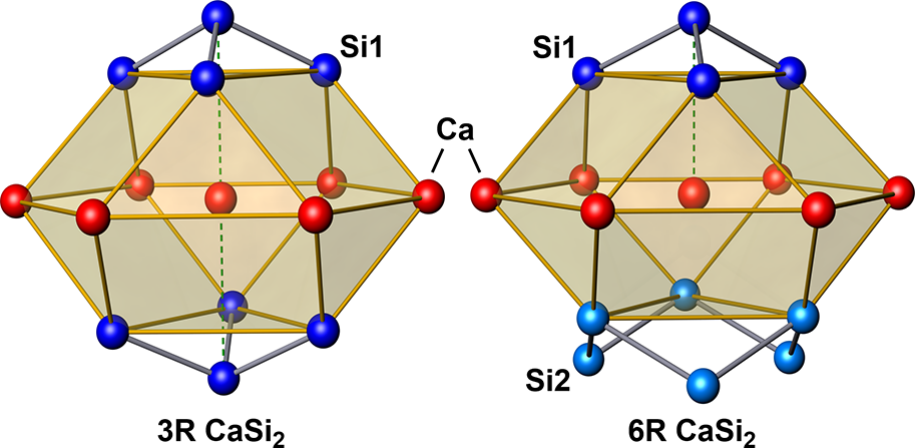
Coordination of the Ca atoms in 3R and 6R-CaSi_2_.

**Figure 8:**
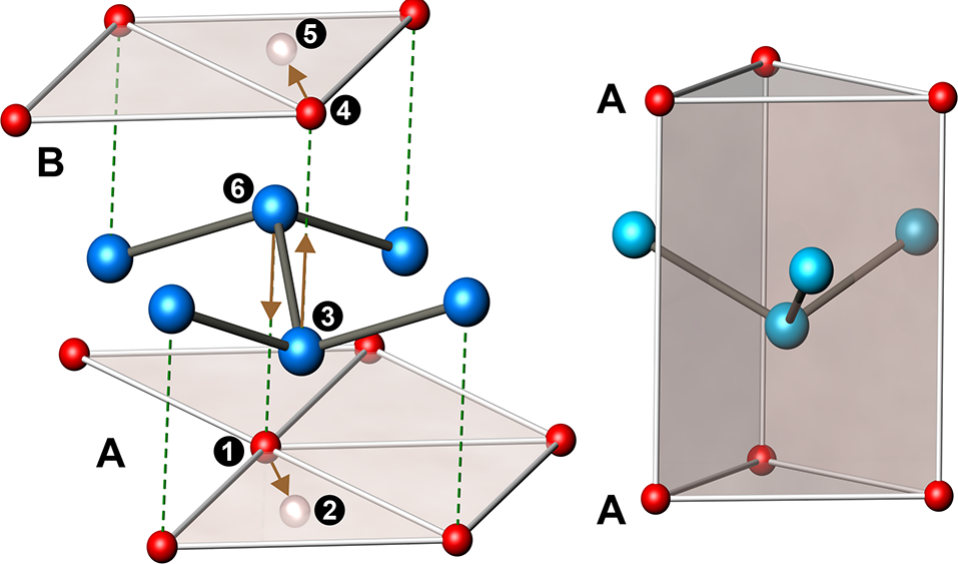
Coordination of Si atoms in the case of AB-stacking (left) and AA-stacking (right) of the Ca layers.

In all polytypes, the Ca atoms occupy the centers of capped anticuboctahedra formed by neighboring Ca and Si atoms (Figure 7). However, depending on the stacking sequence, the atomic environments vary. In 3R-CaSi_2_, the anticuboctahedra have two caps, resulting in a coordination number 12 + 2. On the other hand, in 6R-CaSi_2_, the coordination number of Ca is 12 + 1, with only one additional Si1 atom in the same [001] column. As a result, the Ca atoms in 6R-CaSi_2_ are slightly shifted away from the center toward the open, six-membered ring of Si2 atoms. This shift was found to increase under high pressure.^13^ Similarly, the local environment of the Si atoms also depends on the Ca stacking. When the registry letters of two neighboring Ca layers differ, as in the sequence A *ba* B, the Si atoms of the intervening layers each have an adjacent Ca atom in the same column with a close distance of ≈3 Å (see positions 1 + 6, and 3 + 4 in Figure 8). This coordination pattern applies to the Si1 atoms in 6R-CaSi_2_ and all Si atoms in 3R-CaSi_2_. When there is no registry shift of adjacent Ca layers, as in the sequence A *bc* A, the Si atoms of the intervening layer occupy a trigonal Ca_6_ prism (Figure 8). This arrangement is observed for Si2 in 6R-CaSi_2_ and all Si atoms in 1P-CaSi_2_. Within the Ca_6_-prisms, the Si2 atoms in 6R-CaSi_2_ have more available space along the [001] direction compared to the Si1 atoms, suggesting that the vibration modes of Si2 may be “softer” than those of Si1. In fact, Raman spectroscopy on 6R-CaSi_2_ single crystals revealed *A*_1g_ modes of 368 cm^–1^ for Si1 and 343 cm^–1^ for Si2.^19^ Seemingly, a higher packing density can be achieved by the prism packing, as evidenced by the high-pressure phase 1P-CaSi_2_, which exclusively consists of this motif.

From the crystal structures of the known CaSi_2_ polytypes, three empirical construction rules can be derived:

#### Registry Letters of Adjacent Layers Must Differ

For instance, sequences such as [A *ac* B] or [A *ac* A] with two adjacent atoms A*a* are obviously ruled out for steric reasons. This arrangement would correspond to the occupation of positions 2 + 3 in Figure 8. Consequently, when two adjacent Ca layers have the *same* registry letter, the Si atoms in between must belong to different columns, such as [A *bc* A].

#### Lone Pairs of the Silicon Anions from Adjacent Layers Never Point Directly toward Each Other

**Figure 9:**
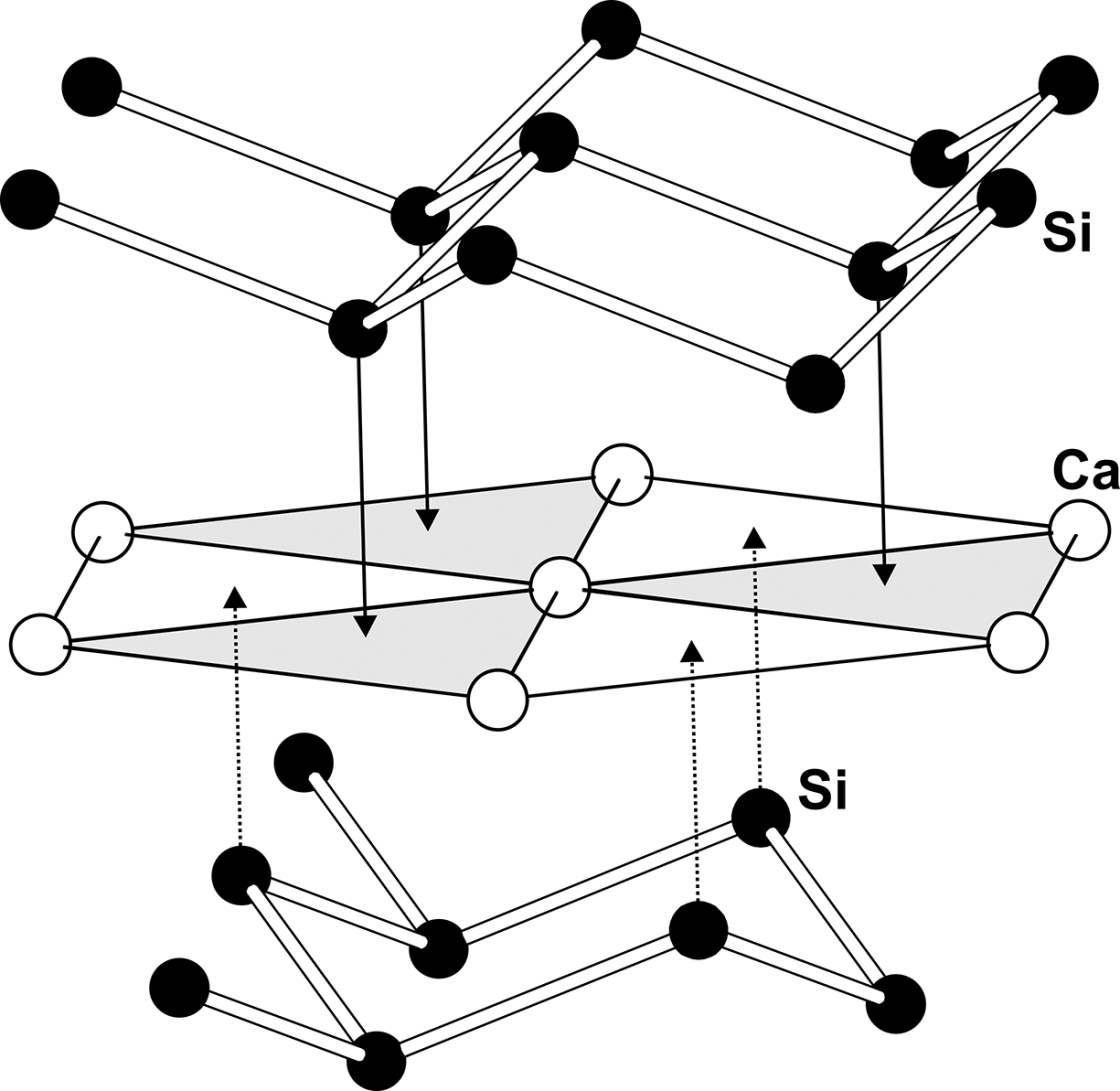
Visualization of the lone-pair rule. Only one lone pair of the (3b) Si^–^ anions points to each triangle of Ca atoms. The arrows represent the direction of the lone pairs.

A sequence such as [*a* B *a* ] is prohibited because the lone pairs assumed for (3b)Si atoms in the “*a”*-layers point to each other. Consequently, in a sequence of one Ca and two Si layers, all registry letters must be different (Figure 9).

#### Ca Layers with Different Registry Letters Enclose Si Layers with the Same Registry Letters but in Reverse Order

A sequence such as [A *ba* B] is allowed, but [A *bc* B] is forbidden. [A *ba* B] allows favorable ionic interactions [A..*a*] and [*b*..B] of Ca and Si atoms on the same rod (atoms 1 + 6 and 3 + 4 in Figure 8). For [A *bc* B], this interaction is missing for the Ca atoms of layer A. Moreover, the lone-pair rule is violated by the forbidden sequence [..*c*B*c*..]:

A *bc* A *ba* B *ca* B *cb* C *ab* C *ac*

A *bc* A *bc* B *ca* B *cb* C *ab* C *ac*

In the hypothetical modification 2H-CaSi_2_ with AB-stacking of the Ca layers,^9^ the crystal structure cannot be constructed without breaking the rules above. In the following examples, either the second or the third rule is broken which makes it plausible that the structure has never been observed so far:

A *b**a*****B*****a**b* A *ba* B *ab* A *ba* B (breaking of rule II)

**A***b***c** B *ac* A *bc* B *ac* A *bc* B (breaking of rule III)

To validate the building rules, we constructed hypothetical defect variants of 6R-CaSi_2_, where the rules are breached, and then calculated the ground state. In the first case, the correct stacking sequence [*a* B ***ca*** B *c*] was replaced by [*a* B ***ac*** B *c*], thereby introducing two violations of the second rule in a sequential manner. The structure model was constructed within space group *P*3*m*1 (no. 156). Although the shortest distances *d*(Ca–Si) and *d*(Si–Si) remained unaffected, the energy of the faulted structure increased by +18.34 meV atom^–1^, thus being significantly higher than that of the regular 6R structure. The stacking fault energy Δ*E*/*S*, where Δ*E* is the total energy difference between the faulted and pristine unit cells and *S* is the area of the unit cell in the (001) plane, was computed to +25.36 meV Å^–2^ or +406.3 mJ m^–2^. In a second defect model, the original sequence [*c* A *ba* B *c*] was replaced by the sequence [*c* A *bc* B *c*], thus violating the second and the third rule. In this case, the energy rose by only +13.96 meV atom^–1^, corresponding to a stacking fault energy of +19.30 meV Å^–2^ or +309.2 mJ m^–2^, respectively. But in both cases, the costs for a 2D defect are so high that only small defect concentrations are expected at lower temperatures. However, at high temperatures, entropy contributions favor defect formation, which may then be preserved during rapid quenching.

### Crystal Structure of 21R-CaSi_2_

The new polytype 21R-CaSi_2_ was identified in HREM images as a stacking variant of 6R-CaSi_2_. Therefore, we assumed that the building rules derived for the other polytypes above also apply to the crystal structure of 21R-CaSi_2_. From the HRTEM images, the positions of the Ca atoms were resolved experimentally (Figure 4), revealing the stacking sequence along [001] as

AABBCC A BBCCAA B CCAABB C.

**Figure 10:**
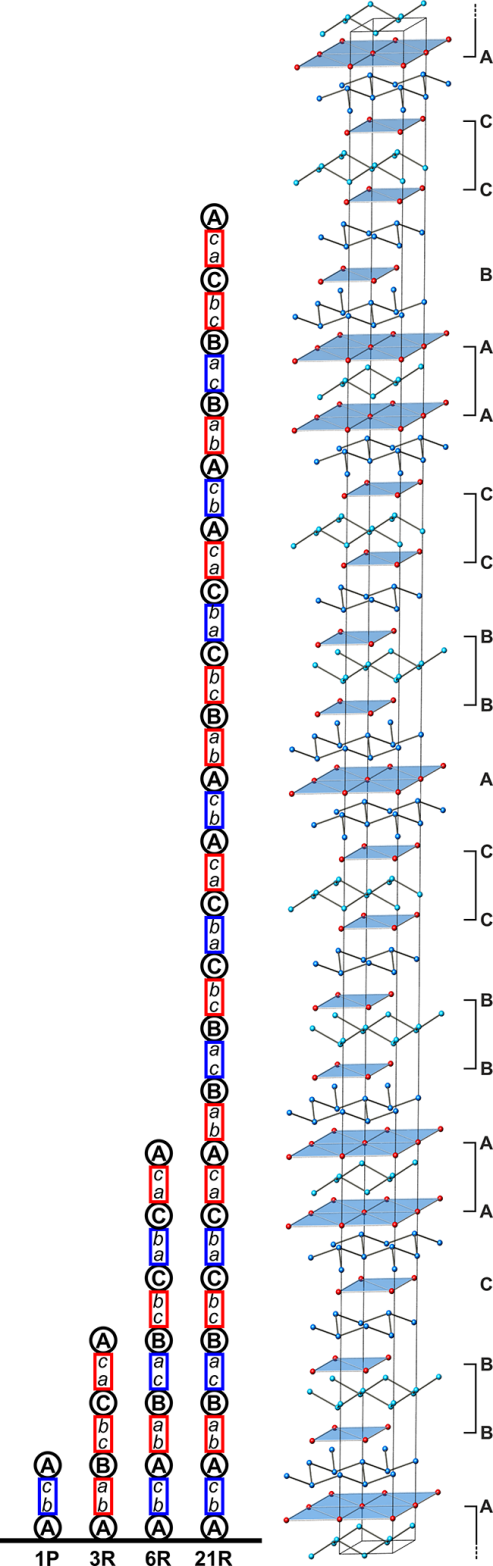
(left) Formal description of the CaSi_2_ polytypes. Registry letters represent the columns [0 0 *l*], [2/3 1/3 *l*], or [1/3 2/3 *l*]. Capital letters represent Ca layers, and lowercase letters represent Si layers. (right) Unit cell of 21R-CaSi_2_. Si atoms are drawn blue, and Ca atoms are red.

In a preliminary structure model, the positions of the Si atoms were derived based on the aforementioned construction principles. The resulting structure model in space group *R*3̅*m* consists of 4 Ca and 7 Si positions and exhibits alternating building blocks with 6R and 3R structure motifs (Figure 10):

A *bc* A *ba* B *ca* B *cb* C *ab* C *ac* A *ba*

B *ca* B *cb* C *ab* C *ac* A *bc* A *ba* B *cb*

C *ab* C *ac* A *bc* A *ba* B *ca* B *cb* C *ac*

The so obtained preliminary atomic coordinates and unit cell parameters were fully optimized using the FPLO code.^27^ The optimized distance values *d*(Si–Si) in 21R-CaSi_2_ revealed ≈2.437 Å for AB-stacking and ≈2.387 Å for AA-stacking, respectively. This phenomenon is observed in all polytypes and might reflect the stronger ionic interactions of Ca and Si atoms when they are adjacent on the same column for AB-stacking of Ca (For detailed crystallographic data, please refer to Tables S1 and S2).

### Stability of the Polytypes

**Figure 11:**
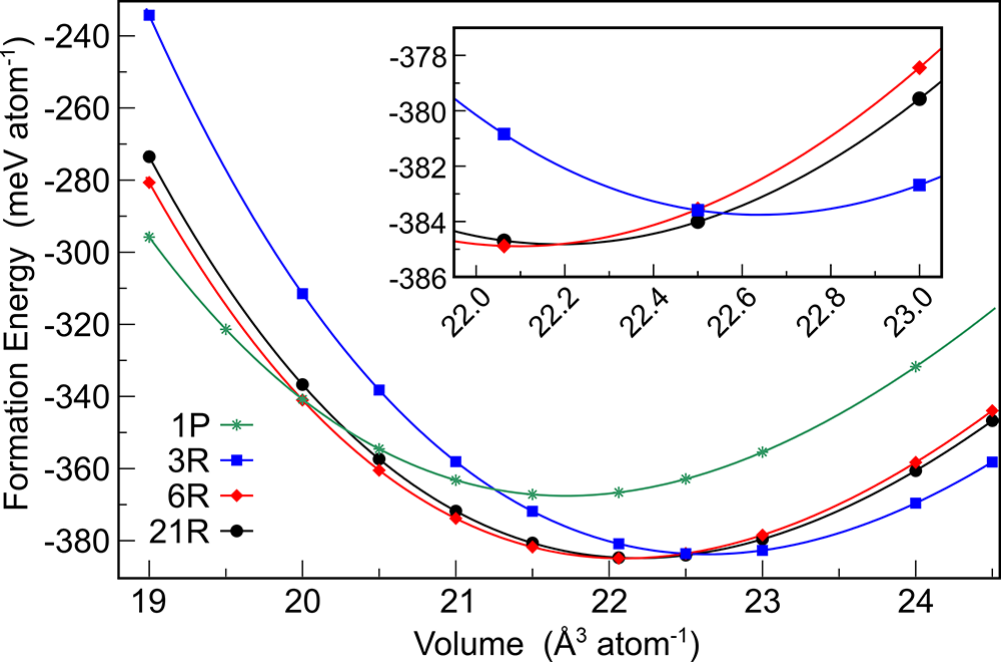
Ground state energy vs volume of the CaSi_2_ polytype formation energies as a function of unit cell volume per atom.

The formation energies for the 3R, 6R, and 21R polytypes were computed with respect to cell volume (Figure 11). Consistent with a previous study,^9^ the 6R structure exhibits the highest formation energy among all polytypes, at 384.89 meV per atom (Table 2). However, the energy difference between the 6R and 21R polytypes of Δ*E*_f_ = 0.07 meV/atom is small and within the margin of error for the total energy calculations. The 6R structure is favored at volumes below 22.20 Å^3^ atom^–1^, while the 3R structure is preferred at volumes larger than 22.56 Å^3^ atom^–1^. In the intermediate range, the 21R structure is stable. The transition from the 6R structure to the 1P structure takes place at a volume of 19.98 Å^3^ atom^–1^, consistent with the experimental observation that 1P-CaSi_2_ is a high-pressure phase. Based on the computed enthalpy values, the stability ranges in pressure are determined as follows: 1P for *p* > 6.32 GPa, 6R for 6.32 GPa > *p* > −0.13 GPa, 21R for −0.13 GPa > *p* > −0.38 GPa, and 3R for *p* < −0.38 GPa, where negative pressure means expansion of the lattice. At low temperatures or under moderate applied compressive pressure (up to about 6.3 GPa), only the 6R structure is expected to exist. However, at high temperatures, the 21R polytype may become stable, which could explain the notable morphology of the microstructure indicating a solid–solid phase transformation on cooling (Figure S3b).

**Table 2:** Formation Energy *E*_f_/meV atom^–1^, Equilibrium Volume *V*_eq_/Å^3^ atom^–1^, Bulk Modulus *B*/GPa, and Its Pressure Derivative (Dimensionless) from Equation of State Analysis

polytype	*E*_f_	*V*_eq_	*B*	d*B*/d*p*
1P	–367.56	21.72	56.1	3.54
3R	–383.76	22.64	61.2	4.06
6R	–384.89	22.10	59.8	4.08
21R	–384.82	22.19	60.0	4.13

The calculated bulk modulus *B* also reflects the stacking of the polytypes (Table 2). The more dominant the AB type compared to the AA type stacking in the crystal structure, the higher is the bulk modulus. We assume that due to the additional steric interaction between Ca and Si atoms along the same column, AB-stacking cannot be compressed as easily as the [SiCa_6_] prisms in an AA-stacking (Figure 8).

### Electronic Structure

**Figure 12:**
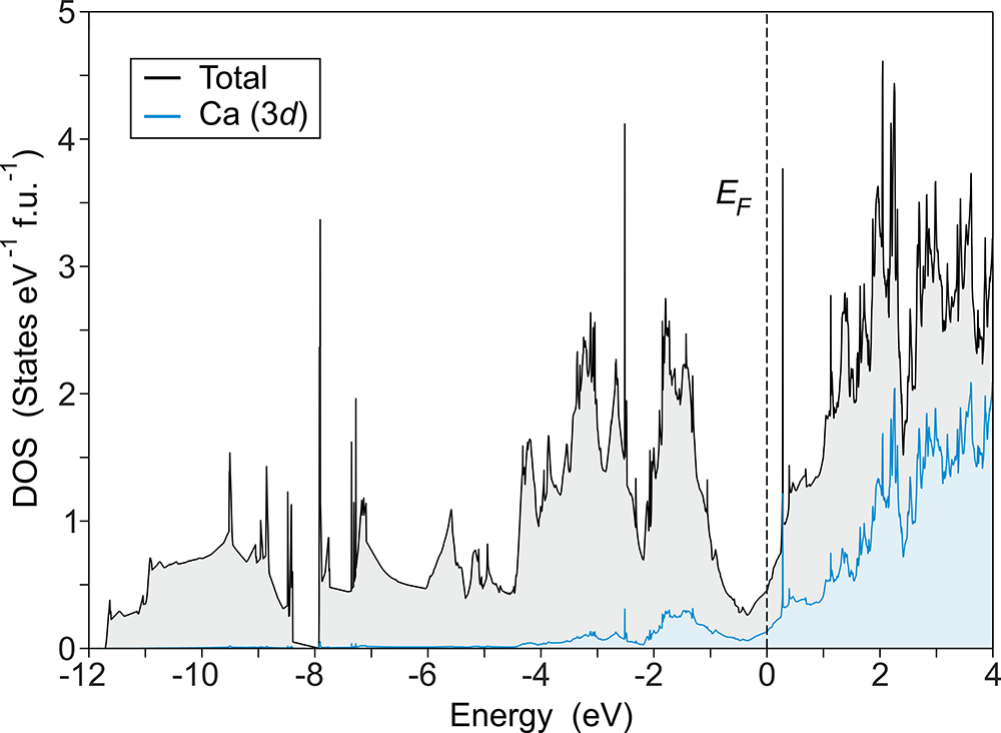
DOS of 21R-CaSi_2_, computed at its theoretical equilibrium structure. Due to the large number of Wyckoff positions in 21-CaSi_2_, the comparison of 3*s* and 3*p* states is presented in Figure S14.

Although the crystal structure of CaSi_2_ corresponds to a typical Zintl phase, quantum chemical calculations^11,44^ and experiments^17,44,45^ in the literature have revealed metallic behavior for all polytypes. The electronic density of states (DOS) revealed a significant contribution of unoccupied Ca *d* states and Si *p* states at the Fermi level. The Fermi level has thus been characterized to consist of Ca *d*-like electrons and Si *p*/Ca *d*-hybridized holes.^11^ This result is not surprising, as the complete charge transfer according to the Zintl rule, while providing a valuable model for structure prediction, should not be misunderstood as the actual charge distribution. Hence, even prototypical Zintl phases such as CaSi^11,46^ and Ba_3_Si_4_^47^ show only partial charge transfer and exhibit metallic properties. In this work, the DOS was computed for the known polytypes and for 21R-CaSi_2_ at their theoretically optimized structures (Tables S1–S8). The calculated DOS for 1P, 3R, and 6R-CaSi_2_ are consistent with those of earlier studies (Figure S15), and also 21R follows the same characteristics (Figures 12 and S16). The Ca 3*d* contributions become significant above ≈ −2 eV and continue to increase through the Fermi energy. As expected, they dominate the unoccupied part of the spectrum.

The Ca–Si interaction is also evident in the analysis of chemical bonding using the Electron Localization Indicator (ELI). The calculations reveal a lone-pair feature at the silicon atoms with ≈1.8 electrons, independent of the stacking order. However, approximately ≈10% of the electron pair is associated with calcium atoms. This confirms Hamann’s description^11^ that the Ca–Si interaction in CaSi_2_ involves the interaction of a Si lone pair with three adjacent Ca atoms.

### Transformation Scenario

While the energetic differences between the polytypes 3R, 6R, and 21 R are small, our structure models of 6R-CaSi_2_ with stacking faults revealed in calculations a significantly higher ground state energy (see above). Therefore, it would be expected that there is an energy barrier for the transformation of the polytypes, which can only be overcome at higher temperatures or after long annealing times. However, this contradicts the following experimental facts:

- The conversion of 6R-CaSi_2_ to 3R-CaSi_2_ in AlCl_3_/toluene takes place at 80 °C.
- The low-temperature conversion leads to a highly crystalline phase.
- DSC measurements of single-phase 3R and rapidly quenched 6R-CaSi_2_ specimens did not exhibit any thermal effect below the melting point. No thermal signal indicating a phase transition was observed.

These observations suggest that the transformation does not involve the breaking of Si–Si bonds. An alternative path is the inversion of the puckered silicon layers, i.e., their reflection at a (001) plane centering each layer. During the inversion, adjacent Si atoms approach each other to a minimum distance of ≈2.2 Å (Figure 6). This minimum distance is distinctly smaller than *d*(Si–Si) = 2.35 Å in α-Si, but could be still sufficiently large for a transition state in combination with local distortions. Experimental evidence confirming the transformation mechanism between 3R-CaSi_2_ and 6R-CaSi_2_ requires in situ studies that are beyond the scope of this work.

**Figure 13:**
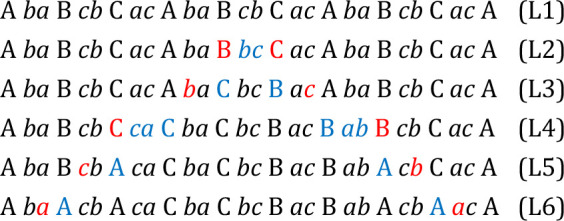
Model for the transformation from 3R- to 6R-CaSi_2_ induced by the inversion of a silicon layer.

However, the inversion of a single Si layer would be sufficient to transform both polytypes into each other, provided that in the vicinity of the inverted layer, the three construction rules are restored in a path of structural rearrangements. This will be demonstrated in the following example for the transformation from 3R-CaSi_2_ to 6R-CaSi_2_. In our model, we assume that only the Ca atoms can switch between the columns [0 0 *l*], [2/3 1/3 *l*], and [1/3 2/3 *l*] during the transformation process. Without breaking their covalent bonds, the Si atoms always remain in their original column and only change their *z*-coordinate. Starting with the stacking sequence of 3R-CaSi_2_ ABCABC (Figure 13; Line L1), an inverted Si layer is introduced between a Ca–B and a Ca–C layer (L2; blue registry letters). To avoid short distances to the Si atoms and violation of rule I, the adjacent Ca atoms (L2; red letters) shift from column B → C and from C → B, respectively (L3; blue letters). However, the sequences [A *ba* C] and [B *ac* A] are forbidden according to rule III (L3; red letters). Again, the violation is resolved by changing the registry of the adjacent Ca layers and inverting the next adjacent Si layers (L4; blue letters). The resulting violation of rule I (L4; red letters) leads to a position change of C → A and B → A for the affected Ca atoms (L5; blue letters). By repeating the steps L3-L4, the stacking order of 6R-CaSi_2_ AABBCC is achieved (L6). In this or a similar manner, the transformation might proceed without breaking Si–Si bonds.

### Role of α-Si

A HRTEM study of 6R-CaSi_2_ layers grown on α-silicon substrates by vapor deposition provides a hint as to why the stability of 3R-CaSi_2_ is promoted by the presence of α-Si.^45^ It was revealed that the growth of CaSi_2_ on a silicon layer always begins with a registry change (AB). A homogeneous distribution of Si precipitates could therefore be the deciding factor in stabilizing 3R-CaSi_2_. It has been reported that 3R-CaSi_2_, formed in an H_2_ atmosphere, no longer transforms into 6R-CaSi_2_ through heat treatment at 800 °C.^9^ From this, we conclude that 3R-CaSi_2_ does not convert into the stable 6R form if a certain concentration of silicon is exceeded, which is a likely scenario after annealing in an H_2_ atmosphere. Nanosheets of silicon (Figure S11b) are not detected in PXRD analysis. This model is supported by the absence of 3R-CaSi_2_ in all samples prepared with Ca excess because an excess of Ca prevents the formation of α-Si slabs.

### Magnetic Susceptibility

In previous studies, structure calculations revealed that CaSi_2_ is metallic,^11^ despite fitting the ionic description of the Zintl concept with an electron balance of [Ca^2+^]3b[Si^–^]_2_. In metallic Zintl phases, the diamagnetic contributions of the elements generally outweigh the Pauli paramagnetism of the conduction electrons. Consequently, both 3R-CaSi_2_ and 6R-CaSi_2_ samples are diamagnetic. The diamagnetism per formula unit is more pronounced for 6R-CaSi_2_ with χ = −20 × 10^–6^ emu/mol, compared to 3R-CaSi_2_ with χ = −5 × 10^–6^ emu/mol. We assume that the different coordination of the Si atoms results in varying diamagnetic contributions. A more comprehensive analysis of the physical properties is planned for a future publication.

## Conclusions

A defect-rich form of polycrystalline 6R-CaSi_2_ has been obtained through a manual splat-cooling technique. Additionally, the new polytype 21R-CaSi_2_ was discovered, representing an intergrowth of 3R-CaSi_2_ and 6R-CaSi_2_. 3R-CaSi_2_ is stabilized by the presence of silicon nanoslabs, which can arise from the oxidation or evaporation of calcium. The transformation from defect-rich 6R-CaSi_2_ to 3R-CaSi_2_ is possible by inversion of the Si layers without breaking Si–Si bonds. Therefore, the structural reorganization occurs under mild conditions and demonstrates reversibility for low concentrations of silicon impurities. Prolonged annealing times ultimately lead to the thermodynamically stable phase of 6R-CaSi_2_.

## Supporting Information Available

Details on preparation, microstructure analysis, EBSD, PXRD, HRTEM, SAED, crystallographic data, and electronic density of states (PDF)
