# Supplementary material for: Deciphering the Polymorphism of CaSi2: The Influence of Heat and Composition
Source: Inorg Chem. 2024 May 24;63(23):10557–67. doi: 10.1021/acs.inorgchem.4c00902 (PMC11167640; doi:10.1021/acs.inorgchem.4c00902)
Supplement: Supplementary file 1 — ic4c00902_si_001.pdf [file ic4c00902_si_001.pdf]

Supporting information:

Deciphering the Polymorphism of $CaSi_2$: The Influence of Heat and Composition.

Xian-Juan Feng, Wilder Carrillo-Cabrera, Alim Ormeci, Mitja Krnel, Ulrich Burkhardt, Bodo Böhme, Yuri Grin, Michael Baitinger

Max-Planck-Institute for Chemical Physics for Solids, 01187 Dresden, Germany.

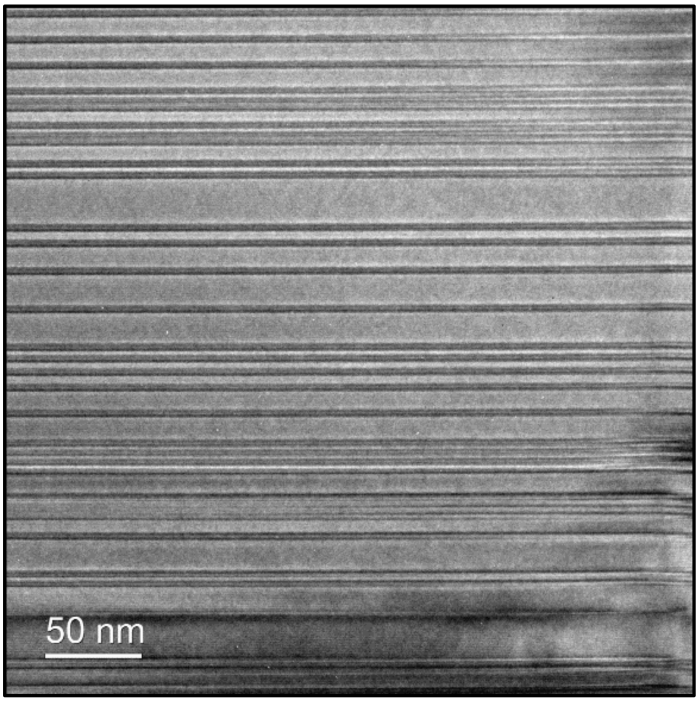

Table of Content

1. **Preparation of 6R $CaSi_2$ by rapid cooling**
2. **Microstructure analysis**
3. **EBSD Investigations**
4. **High resolution transmission electron microscopy and electron diffraction**
5. **PXRD measurments**
6. **Crystallographic data from quantum chemical structure optimization**
7. **Electronic density of states**
8. **References**

1. Preparation of 6R $CaSi_2$ by rapid cooling

6R-$CaSi_2$ was obtained by rapid quenching of a stoichiometric melt. After melting in a glassy carbon crucible, the sample was poured onto a steel plate and cooled down by punching the droplet with a second plate. Typically, flakes and pieces with a thickness of $d \approx 1$ mm were obtained (Figure S1). The cooling rate is faster than 1000K/sec because the flakes can be touched directly after punching. A detailed illustration of the manual splat cooling procedure has been published.[1]

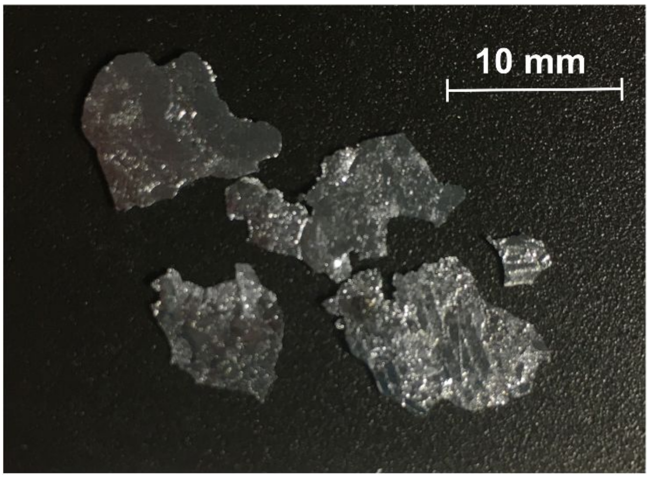

Figure S1: Flakes of 6R-$CaSi_2$ obtained from manual splat cooling in an argon atmosphere. The photo was taken on air, where the surfaces became tarnished after an hour.

2. Microstructure analysis

2.1 As cast 6R-$CaSi_2$

Platelets obtained from rapid quenching were polished on air. SEM images revealed crystalline grains of up to 100 µm in size (Figure S2). The composition $CaSi_2$ was confirmed by EDXS.

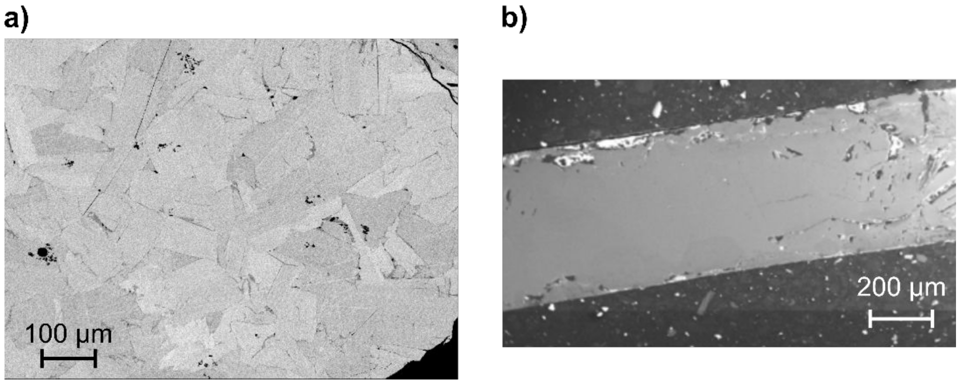

Figure S2: SEM images of a 6R-$CaSi_2$ platelet obtained from manual splat cooling: a) top view, BSE contrast, b) cross-section, SE contrast. Polishing on air with SiC abrasive paper introduced black spots and cracks.

In optical microscopy, bright-field images also revealed homogeneous samples, with minimal presence of cracks (Figure S3a). Polarized light images unveiled bands of varying thicknesses, typically indicating twinned specimens, antiphase boundaries or 2D defects such as changes in stacking disorder (Figure S3b).

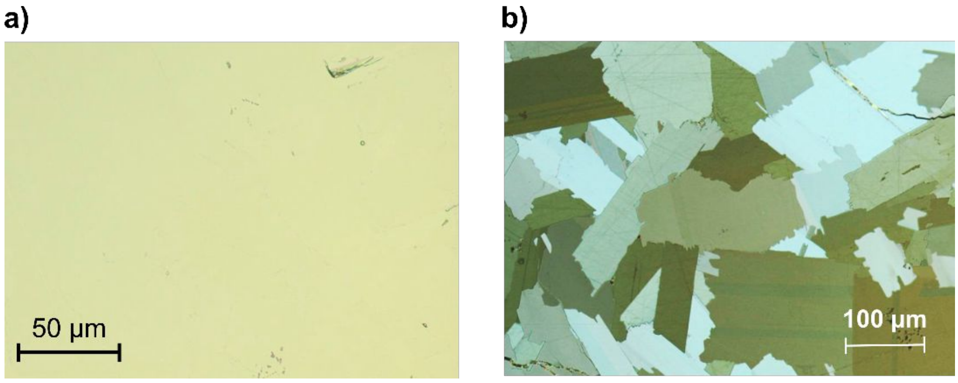

Figure S3: Optical microscopy images of active 6R-$CaSi_2$ obtained from manual splat cooling: a) Bright field image indicating phase purity; b) Polarized light image revealing bands crossing the crystallites. Contrast was enhanced using graphic software.

2.2 3R-CaSi$_2$

Microstructure analysis was conducted on platelets of 3R-CaSi$_2$ obtained through annealing at 600 °C. The sample presented in the following was confirmed to be single-phase according to PXRD. Optical microscopy images of 3R-CaSi$_2$ (Figure S4) showed more cracks and noticeably smaller grains than the 6R samples (Figure S2) prior to annealing. The stripe features observed in 6R-CaSi$_2$ were largely absent. The fine-grained structure and a large number of cracks are also revealed by SEM (Figure S5).

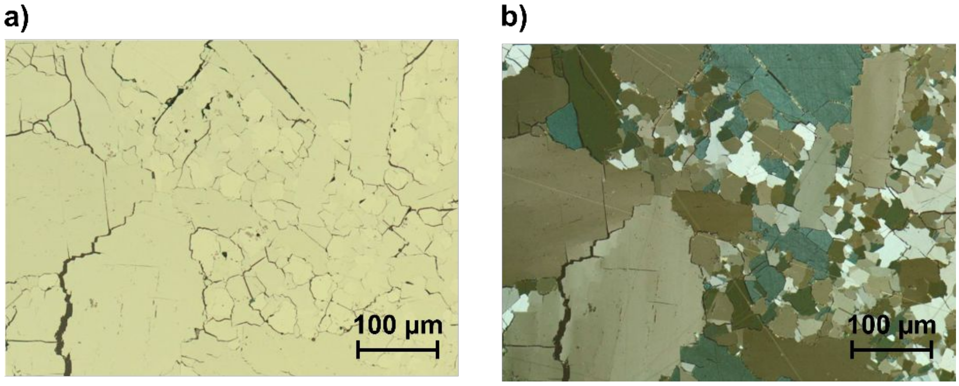

Figure S4: Optical microscopy images of 3R-CaSi$_2$ obtained from annealing reactions: a) bright field image indicating the phase purity; b) polarized light image.

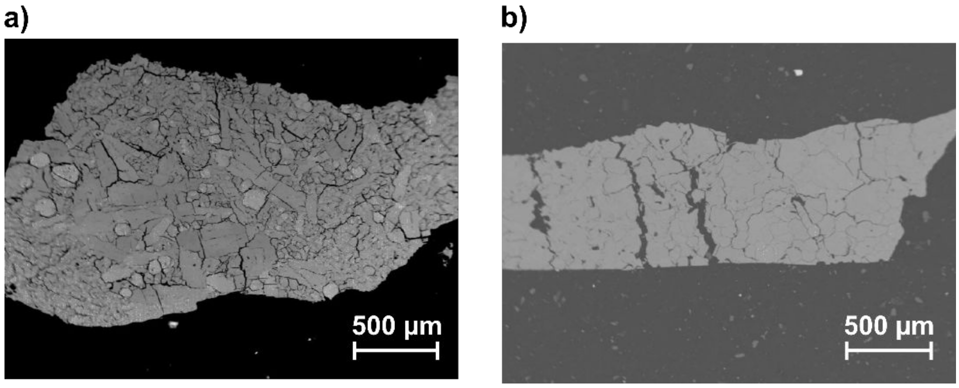

Figure S5: SEM images of a platelet consisting of 3R-CaSi$_2$ obtained from annealing: a) top view, BSE contrast, b) cross-section, BSE contrast.

3. EBSD Investigations

The crystal structures at the sample surface were identified by electron backscattering diffraction (EBSD) in a scanning electron microscope (SEM). Three samples are presented: After rapid quenching, the surface of the specimens solely comprised 6R-$CaSi_2$ (Figure S6a), consistent with the XRPD analysis of the bulk phase. By annealing at 600°C, as-cast samples of 6R-$CaSi_2$ transformed to 3R-$CaSi_2$. EBSD analysis of a sample, where the conversion was still incomplete according to XRPD, revealed a single-phase surface of 3R-$CaSi_2$ (Figure S6b). Hence, the transformation was completed earlier at the surface than in the bulk material, likely initiating at the surface. When the transformation from as-cast 6R-$CaSi_2$ to 3R-$CaSi_2$ was complete in the bulk phase, as indicated by XRPD, the sample surface once again exhibited areas of 6R-$CaSi_2$, indicating the onset of back transformation (Figure S6c). Simulated Kikuchi patterns for 3R- and 6R-$CaSi_2$, used in the EBSD analysis, are exemplarily shown perpendicular to the [001] direction (Figure S7). These patterns illustrate the threefold symmetry of 3R-$CaSi_2$ and 6R-$CaSi_2$ but show with notable differences in the intensities of the Kikuchi lines.

a) b) c)

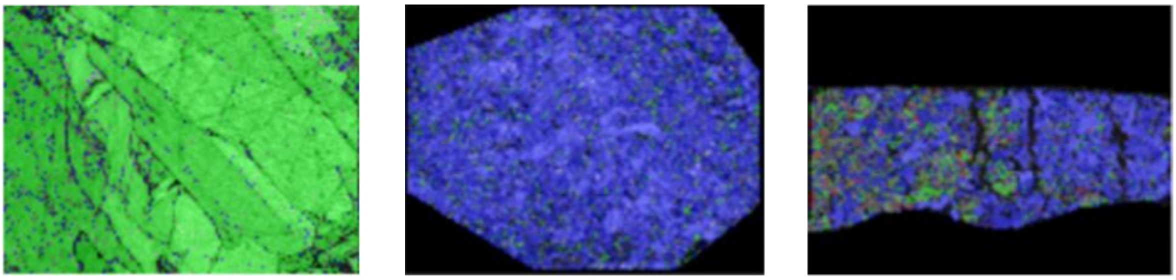

Figure S6: EBSD images of $CaSi_2$ platelets prepared under different conditions. The horizontal side of the images show approximately 2 mm of the sample. Green areas denote regions of 6R-$CaSi_2$; blue areas represent 3R-$CaSi_2$. a) samples obtained from manual splat cooling, b) after annealing for 20 hours, c) annealing for 24 hours.

a) b)

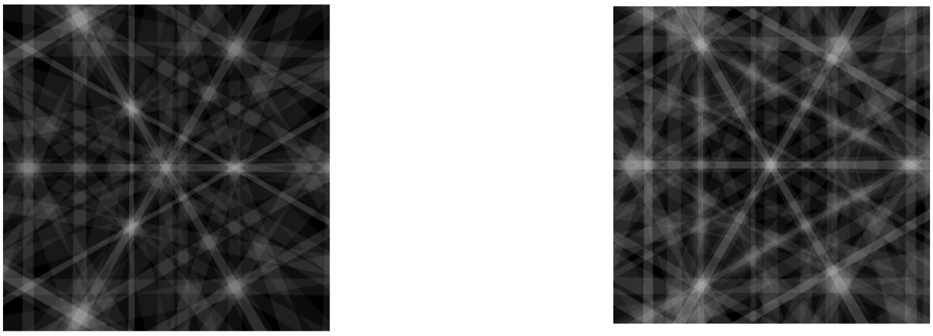

Figure S7: Kiguchi diffraction patterns along [001] of a) 3R-$CaSi_2$ and b) 6R-$CaSi_2$.

4. High resolution transmission electron microscopy and electron diffraction

For a TEM and electron diffraction study, thin lamella specimens were prepared with the focused ion beam technique (FEI, Eindhoven) in a Quanta 200 3D ion/electron dual-beam device using the lift-out technique. The samples were cut perpendicular to the basal plane of the platelets (Figure S1), followed by thinning of the cross sections. The samples were further investigated by combining transmission electron microscopy (conventional and HRTEM) and selected area electron diffraction (SAED). The latter investigations were performed on an FEI Tecnai F30-G2 super-twin microscope operating at 300 kV equipped with a CCD camera (GATAN Inc.) and a standard double-tilt holder (GATAN Inc.).

4.1 Investigations on as-cast 6R-$CaSi_2$

A bright field TEM image of an as-cast 6R-$CaSi_2$ sample indicates both areas free of defects (Figure S8a, region B) and areas showing stacking faults (Figure S8a, region C). For the region free of defects, selected-area electron diffraction patterns revealed a regular diffraction pattern of 6R-$CaSi_2$ with the reflection conditions $-h+k+l = 3n$ for hkl and $l = 3n$ for $00l$, which is in agreement with a triple hexagonal R-cell (Figure S8b). The SA-ED image of the disordered region (Figure S8c) showed the same reflections but, in addition, diffuse lines connecting the reflections along the [001]* direction. These diffuse lines are produced by stacking faults along [001] or antiphase boundaries perpendicular to [001].

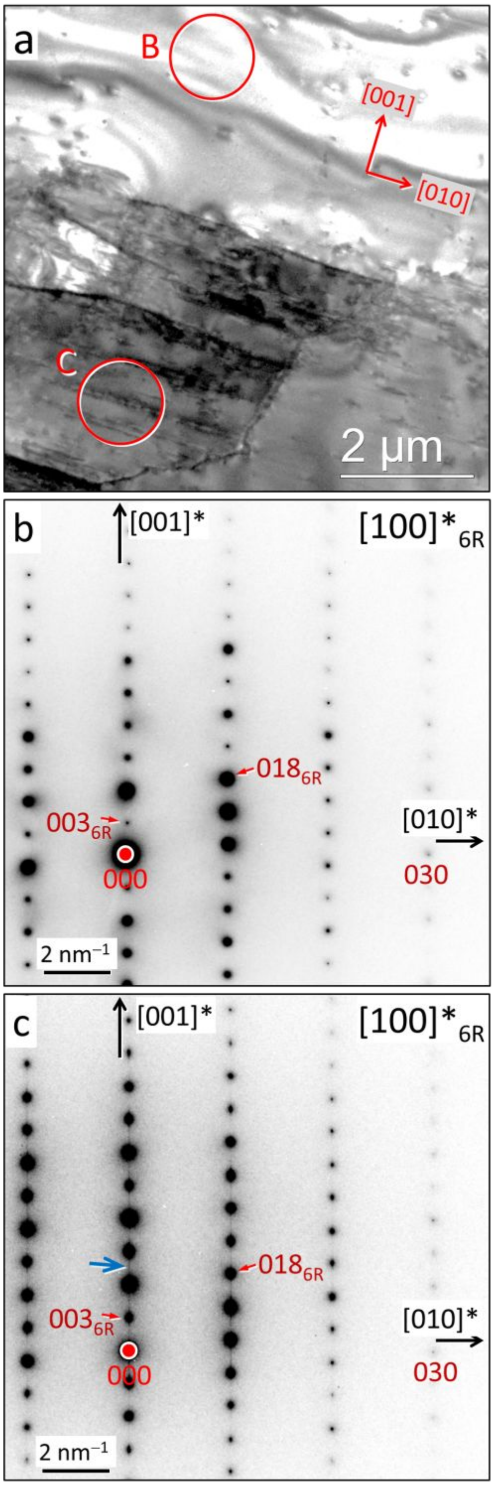

Figure S8. (a) Bright-field TEM image (diffraction contrast) for a sample of as-cast 6R-$CaSi_2$. (b) SAED pattern of region B with ordered crystal structure and c) of region C with defects. The diffuse lines (blue arrow) between the reflections along the [001]* direction point to stacking disorder.

4.2 Investigations on annealed 3R-$CaSi_2$

Samples annealed for 2 days at 600 °C were found to be single-phase 3R-$CaSi_2$ from XRPD analysis. The bright-field TEM image (diffraction contrast) reveals dark linear features and small dark spots within a matrix phase of 3R-$CaSi_2$ (Figure S9a). SA-ED patterns of region B solely revealed a regular diffraction pattern corresponding to the main phase 3R-$CaSi_2$ (Figure S9b). SA-ED patterns of region A, covering black linear features, revealed both the diffraction pattern of 3R- and 6R-$CaSi_2$ (Figure S10), indicating that the linear dark features correspond to the 6R-$CaSi_2$ structure.

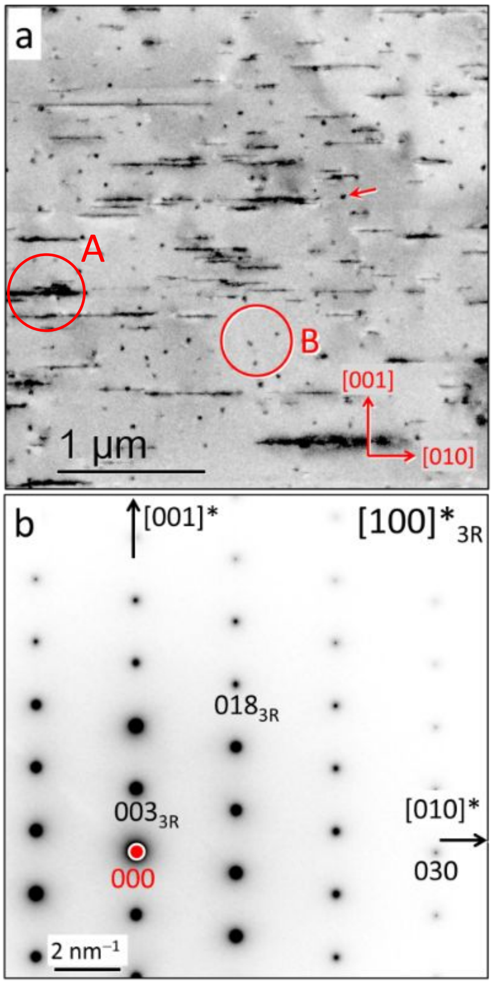

Figure S9. a) TEM bright field image of a 3R-$CaSi_2$ sample revealing linear features and spots in diffraction contrast. b) The SA-ED pattern of region B reveals the expected reflections for the 3R polytype.

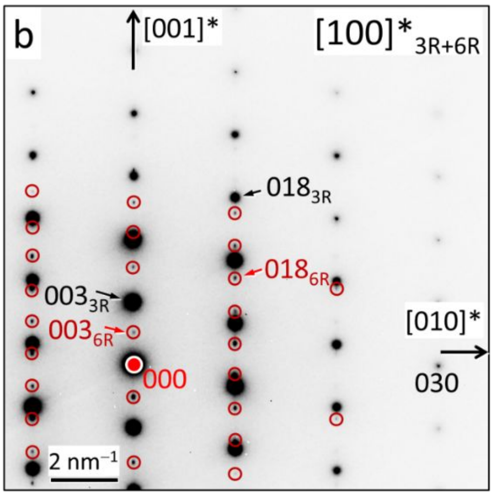

Figure S10. SA-ED pattern of the region A in Figure S9a, showing ED patterns of both 3R and 6R polymorphs.

High-resolution TEM (along [100] direction) around one short linear defect in **Figure S11a** revealed a thin slab of 20.8 Å which appears to be a fragment of the 6R-$CaSi_2$ polymorph. A second kind of defect detected was an 8.4 Å thin slab with composition '$CaSi_4$' (**Figure S11b**) and is probably compensating the excess of silicon, which is therefore not detected in PXRD.

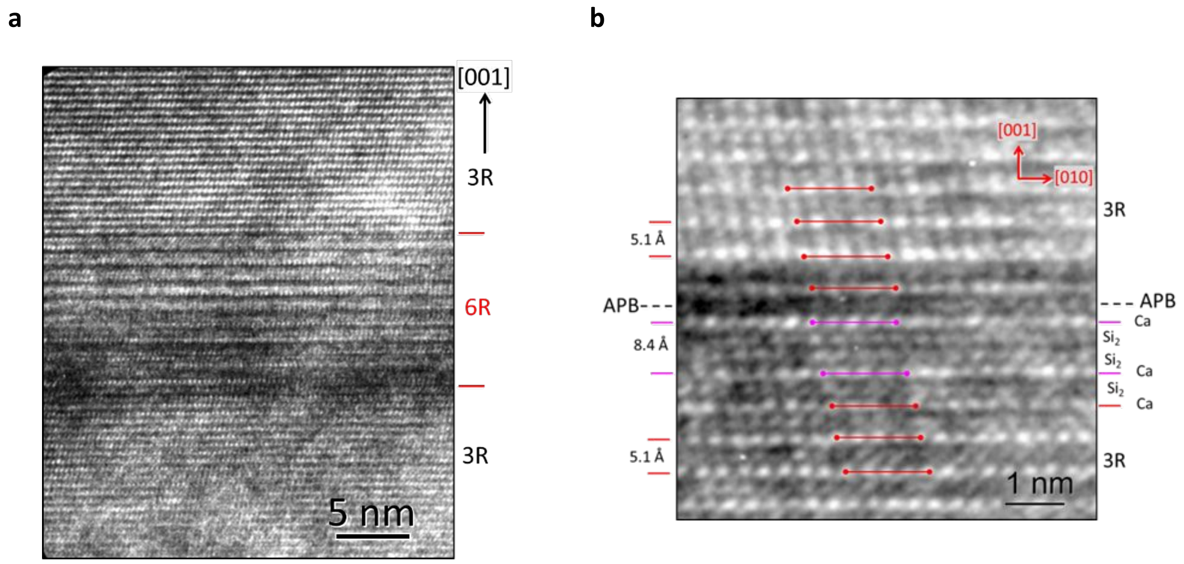

Figure S11. a) High-resolution TEM image (along [100] direction) around one dark linear feature in Figure S9a that appears to be a residual fragment of the 6R-$CaSi_2$ polymorph. b) Another type of linear defect with missing Ca layer and local composition $CaSi_4$.

The dark spots in Figure S9a did not reveal a different crystal structure in HRTEM (Figure S12).

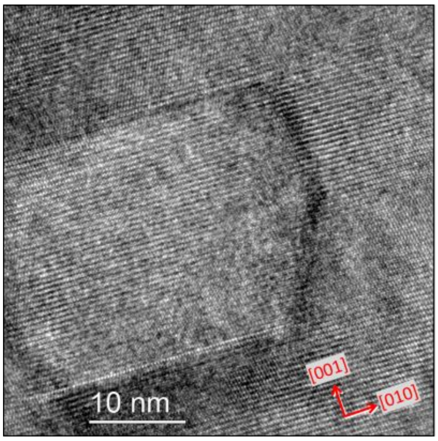

Figure S12. High-resolution TEM image (along [100] direction) of a dark spot feature in Figure S9a. The spots are empty cavities with internal habitus and do not reveal a different crystalline phase.

5. PXRD measurements

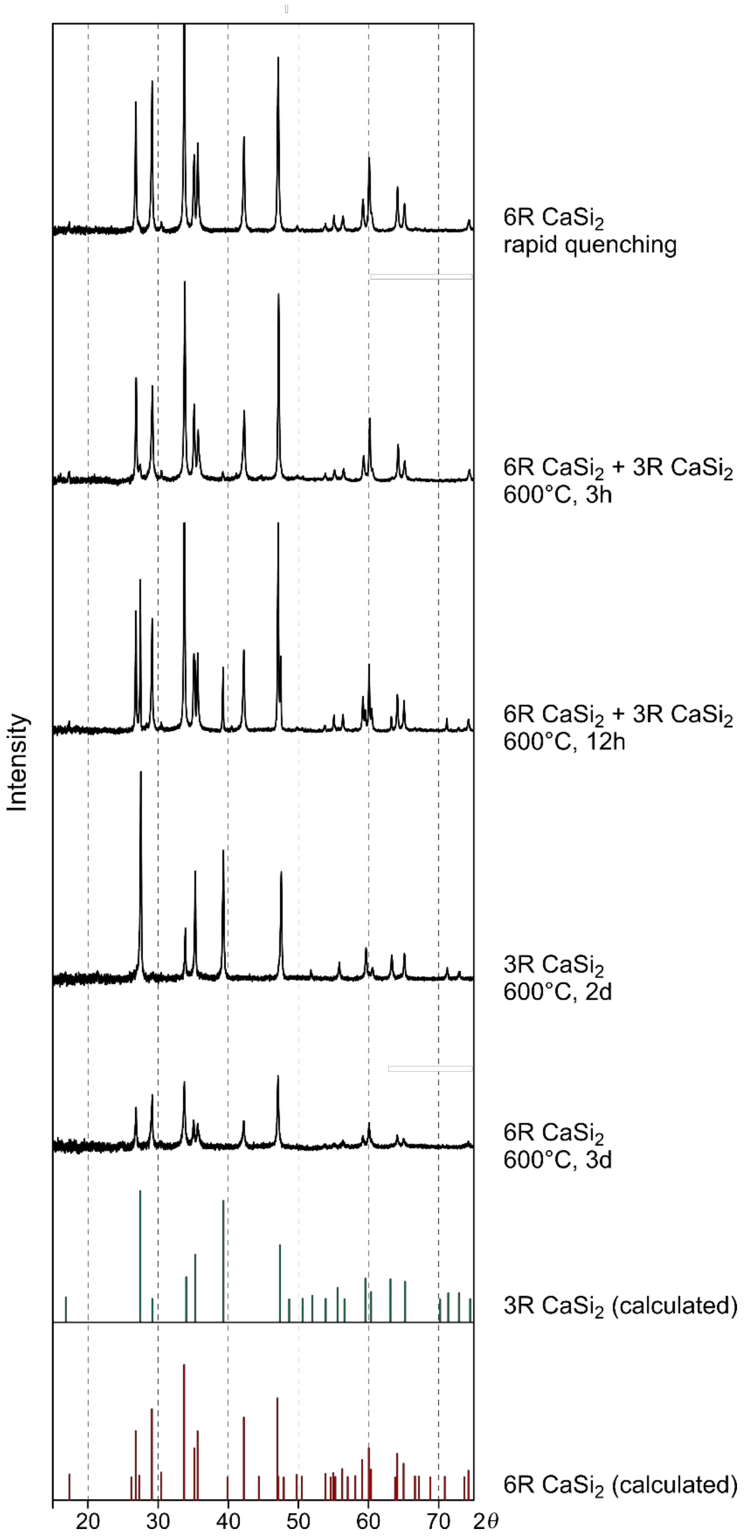

Figure S13. Transformation of 6R $CaSi_2$, prepared by manual split cooling, through heat treatment at 600°C. The transformation times depend heavily on the morphology of the starting material and can vary by a factor of 2.

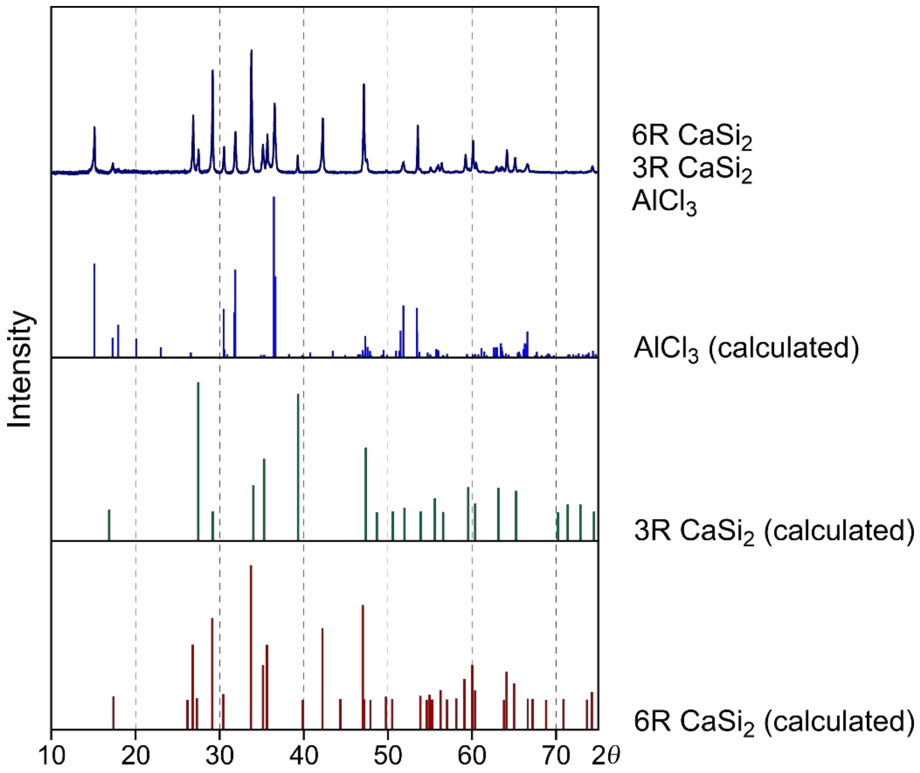

Figure S14. Transformation of 6R $CaSi_2$, prepared by manual split cooling, in a solution of $AlCl_3$ in toluene at 80°C. The diagram shows the product before washing and contains excess $AlCl_3$ that has not dissolved in toluene.

6. Crystallographic data from quantum chemical structure optimization

6.1 21R-$CaSi_2$

Table S1. Crystallographic data of 21R-$CaSi_2$ calculated with with FPLO.[2]

Composition; *Z*	$CaSi_2$; 21
Mole mass	2021.229 g·mol^{-1}
Space group	$R\bar{3}m$ (no.166)
Lattice parameter	*a* = 3.86798 Å
	c = 107.27629 Å
Volume	1389.962 Å^3
Pearson symbol	*hR*63
Calculated density	2.415 g/cm^3
Calculation	FPLO

Table S2. Atomic coordinates of 21R-$CaSi_2$.

Atom	site	*x/a*	*y/b*	*z/c*
Ca1	6*c*	0	0	0.022848
Ca2	6*c*	0	0	0.215844
Ca3	6*c*	0	0	0.261424
Ca4	3*b*	0	0	1/2
Si1	6*c*	0	0	0.051924
Si2	6*c*	0	0	0.098663
Si3	6*c*	0	0	0.186708
Si4	6*c*	0	0	0.290509
Si5	6*c*	0	0	0.337282
Si6	6*c*	0	0	0.424137
Si7	6*c*	0	0	0.470872

6.2 1P-$CaSi_2$

Table S3. Crystallographic data of 1P-$CaSi_2$ calculated with with FPLO.[2]

Composition; Z	$CaSi_2$; 1
Mole mass	96.249 g·mol^{-1}
Space group	$P\bar{3}m1$ (no.164)
Lattice parameter	a = 3.8935 Å
	c = 4.9627 Å
Volume	65.152 Å^3
Pearson symbol	*hP*3
Calculated density	2.453 g/cm^3
Calculation	FPLO

Table S4. Atomic coordinates of 1P-$CaSi_2$.

Atom	site	*x/a*	*y/b*	*z/c*
Ca1	1*a*	0	0	0
Si1	2*d*	1/3	2/3	0.5872

6.3 3R-$CaSi_2$

Table S5. Crystallographic data of 3R-$CaSi_2$ calculated with with FPLO.[2]

Composition; Z	$CaSi_2$; 3
Mole mass	288.747 g·mol^{-1}
Space group	$R\bar{3}m$ (no.166)
Lattice parameter	a = 3.8449 Å
	c = 15.9140 Å
Volume	203.741 Å^3
Pearson symbol	*hR*3
Calculated density	2.353 g/cm^3
Calculation	FPLO

Table S6. Atomic coordinates of 3R-$CaSi_2$.

**Atom**	**site**	***x/a***	***y/b***	***z/c***
Ca1	3*a*	0	0	0
Si1	6*c*	0	0	0.1975

6.4 6R-$CaSi_2$

Table S7. Crystallographic data of 6R-$CaSi_2$ calculated with with FPLO.[2]

Composition; *Z*	$CaSi_2$; 6
Mole mass	577.494 g·mol^{-1}
Space group	$R\bar{3}m$ (no.166)
Lattice parameter	*a* = 3.8768 Å
	c = 30.5103 Å
Volume	397.122 Å^3
Pearson symbol	*hR*63
Calculated density	2.415 g/cm^3
Calculation	FPLO

Table S8. Atomic coordinates of 6R-$CaSi_2$.

Atom	site	*x/a*	*y/b*	*z/c*
Ca1	6*c*	0	0	0.0803
Si1	6*c*	0	0	0.1826
Si2	6*c*	0	0	0.3471

7. Electronic density of states

Electronic densities of states (DOS) were computed for the $CaSi_2$ polytypes 1P, 3R, 6R and 21R at their theoretical equilibrium structures (Tables S1-S8).[2] For the analysis, the polytypes 1P- and 3R-$CaSi_2$ are particularly interesting because they each exhibit a uniform stacking variant. Therefore, the DOS of 1P-$CaSi_2$ (Figure S15a) and 3R-$CaSi_2$ (Figure S15b) exhibit characteristic footprints for the two fundamental types of Ca – Si stacking orders:

1P-$CaSi_2$: The Si layers are always situated between Ca atoms with the same registry type, [A *bc* A].

3R-$CaSi_2$: The Si layers are always situated between Ca atoms with different registry type, [A *ba* B].

In 1P-$CaSi_2$, the lowest group of states covers the energy range –8.49 and –11.65 eV, while for 3R-$CaSi_2$, the range spans from –7.91 to –11.25 eV. Accordingly, this range is slightly larger for 3R-$CaSi_2$ (by ≈ 0.2 eV), and shifted to higher energies (by ≈ 0.4 eV) compared to 1P-$CaSi_2$. The range is primarily dominated by Si(3*s*) contributions. Si(3*p*) states also contribute significantly, while Ca contributions are negligible.

The next region in the DOS is initially equally dominated by Si(3*s*) and Si(3*p*) contribitions, but at higher energies up to the Fermi level, it is almost exclusively dominated by Si(3*p*) orbitals. For 3R-$CaSi_2$, the Si(3*p*) orbitals become dominant at –5.3 eV, for 1P-$CaSi_2$ at –4.5 eV. These characteristic footprints are also identifiable in the DOS of 6R-$CaSi_2$ (Figure S15c) and 21R-$CaSi_2$ (Figure S16), where both types of stacking orders, AA and AB, are present. The

primary reason for these shared characteristics in the electronic structure is that the interatomic distances remain consistent across all these polytypes.

The Ca contributions also exhibit highly similar behavior across all polytypes. The Ca(3*d*) pDOS becomes significant at approximately –2 eV and continues to rise through the Fermi energy. As expected, they predominate in the unoccupied part of the spectrum. In regard to integrated projected pDOS values, the occupancies of Ca(4*s*) and Ca(4*p*) are comparable to each other and vary within a narrow range, from 0.27 to 0.31 electrons, while Ca(3*d*) occupancies range between 0.52 and 0.54 electrons.

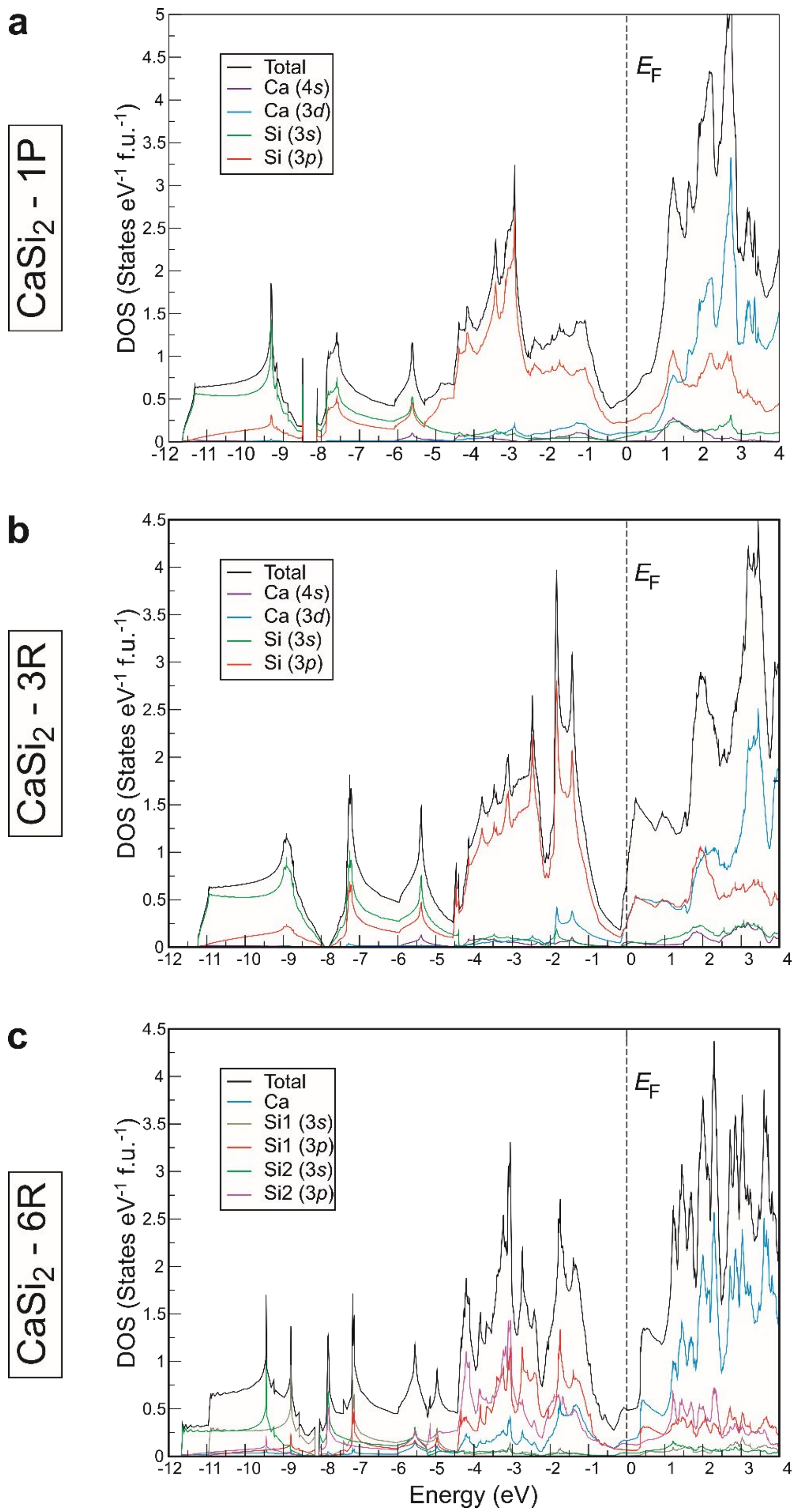

Figure S15. Electronic DOS for (a) 1P-$CaSi_2$, (b) 3R-$CaSi_2$, and (c) 6R-$CaSi_2$, computed for their theoretical equilibrium structures.

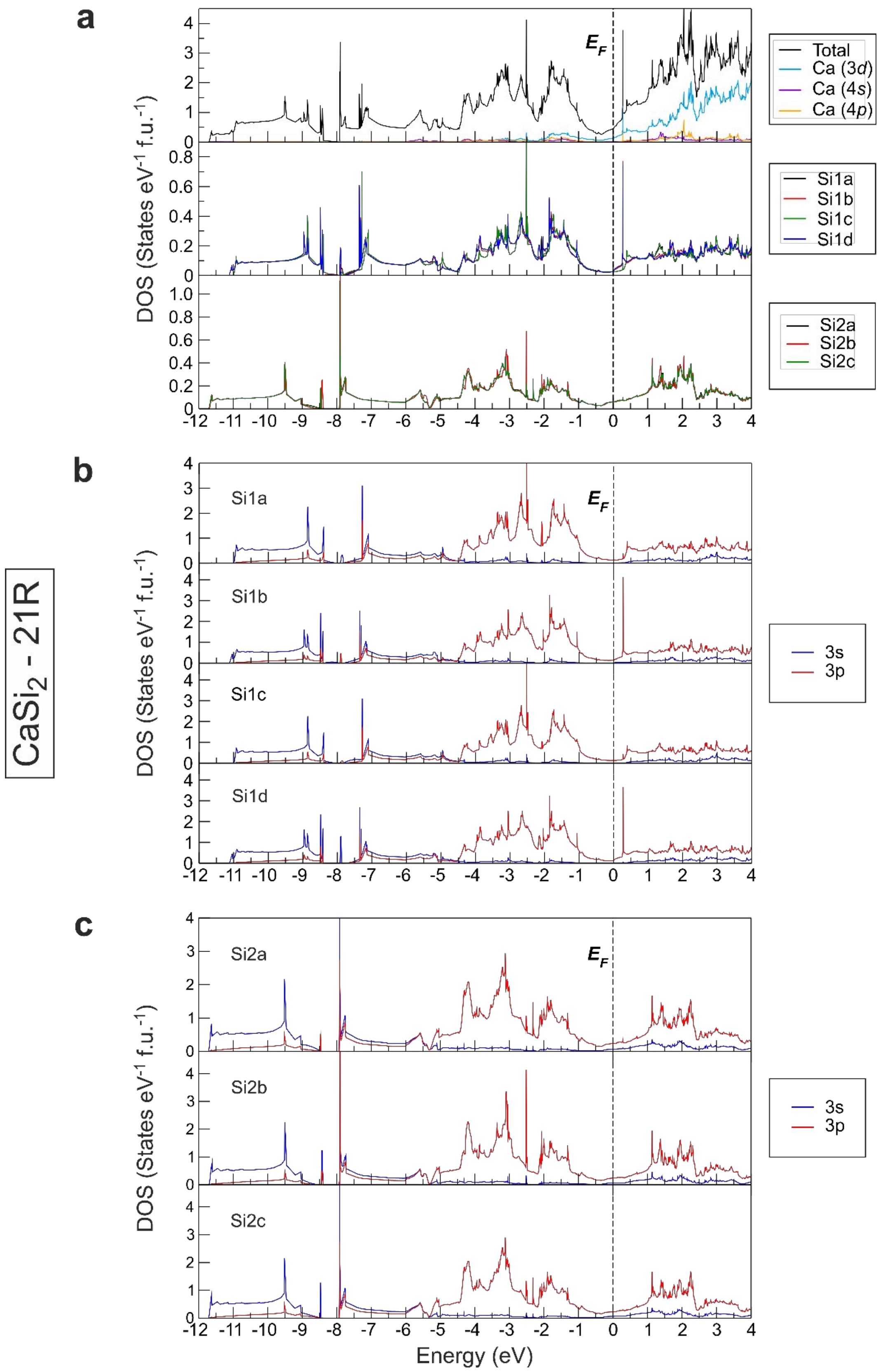

Figure S16. Electronic DOS of 21R-$CaSi_2$, computed at its theoretical equilibrium structure. Due to the large number of Wyckoff positions, the corresponding Si(3*s*)– Si(3*p*) states are presented separately.

8. References

[1] Baitinger, M.; Böhme, B.; Ormeci, A.; Grin, Yu. Solid State Chemistry of Clathrate Phases: Crystal Structure, Chemical Bonding and Preparation Routes. In The Physics and Chemistry of Inorganic Clath-rates; Nolas, G.S., Ed.; Springer Series in Materials Science, Vol. 199; 2014; pp 55-56. DOI: 10.1007/978-94-017-9127-4

[2] Koepernik, K.; Eschrig, H. Full-potential nonorthogonal local-orbital minimum-basis band-structure scheme. *Phys. Rev. B* **1999**, *59*, 1743-1757.
